# Scattering *versus* fluorescence self-quenching: more than a question of faith for the quantification of water flux in large unilamellar vesicles?†

**DOI:** 10.1039/d1na00577d

**Published:** 2021-10-18

**Authors:** Johann Wachlmayr, Christof Hannesschlaeger, Armin Speletz, Thomas Barta, Anna Eckerstorfer, Christine Siligan, Andreas Horner

**Affiliations:** From the Institute of Biophysics, Johannes Kepler University Linz Gruberstr. 40 4020 Linz Austria andreas.horner@jku.at

## Abstract

The endeavors to understand the determinants of water permeation through membrane channels, the effect of the lipid or polymer membrane on channel function, the development of specific water flow inhibitors, the design of artificial water channels and aquaporins for the use in industrial water filtration applications all rely on accurate ways to quantify water permeabilities (*P*_f_). A commonly used method is to reconstitute membrane channels into large unilamellar vesicles (LUVs) and to subject these vesicles to an osmotic gradient in a stopped-flow device. Fast recordings of either scattered light intensity or fluorescence self-quenching signals are taken as a readout for vesicle volume change, which in turn can be recalculated to accurate *P*_f_ values. By means of computational and experimental data, we discuss the pros and cons of using scattering *versus* self-quenching experiments or subjecting vesicles to hypo- or hyperosmotic conditions. In addition, we explicate for the first time the influence of the LUVs size distribution, channel distribution between vesicles and remaining detergent after protein reconstitution on *P*_f_ values. We point out that results such as the single channel water permeability (*p*_f_) depend on the membrane matrix or on the direction of the applied osmotic gradient may be direct results of the measurement and analysis procedure.

## Abstract 2

Accurate *p*_f_ values are of utmost importance to understand the structure–function relationship of water permeation through membrane channels, guiding the design of artificial or biological water channels for separation applications.

## Graphical abstract

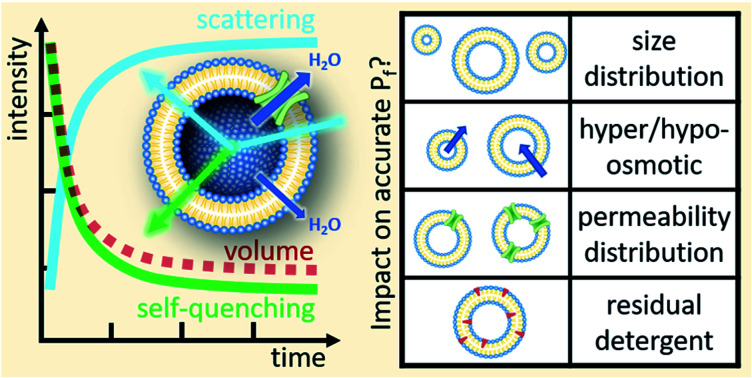

## 1. Introduction

Water homeostasis is of fundamental importance for life and plays a major role in human health and disease, plant growth and bacterial survival. Thereby, the permeation of water is driven by osmotic imbalances of solutes and occurs in either an unfacilitated fashion through the cell membrane or in a facilitated one through membrane spanning protein channels. Classical highly selective water channels, like aquaporins (AQPs), facilitate rapid water transport with approx. 10^9^ water molecules per second. Additionally, different classes of membrane proteins like transporters,^1^ ion channels^2^ and receptors^3^ facilitate passive water flux and may assist or substitute AQPs depending on their site of expression. AQP function itself can be influenced by diverse factors. *E.g.* mutations causing alterations in their expression level, cellular localization, folding or permeability, are directly attributed to human diseases.^4,5^ Furthermore, lipid–protein interactions determine the stability^6^ and function^7^ of transmembrane proteins (MP). Thereby, protein conformation can be influenced by local interactions at the lipid–protein interface^7^ or global interactions such as the lateral pressure profile of the membrane,^8–11^ which in turn is influenced by the lipid composition of the membrane.^12,13^ It has already been shown that lipid-binding at weak interfaces stabilizes loosely interacting oligomers and altering the lipid composition propagates changes in the overall oligomeric state.^6,14^ Besides their substantial role in mammals, AQPs fulfill pivotal functions in plants, where they are also involved in the regulation of cellular water homeostasis.^15^ This includes a key role in transpiration sensitivity to soil drying as well as to high atmospheric vapor pressure deficit (VPD).^16^ Therefore, they represent the perfect target to address abiotic stresses like drought through genetic engineering.^17^ Hence, it is vital to understand the molecular determinants of water transport in order to optimize their performance *in vivo*. Recent progress in the quantification of water flux through narrow MPs^18,19^ already identified the number of hydrogen bonds that water molecules may form with channel lining residues as the major determinant of single-file water transport.^19^ Positively charged amino acids at the pore mouth potentially decrease the dehydration penalty of water molecules entering the single-file region and thereby enhance the passive water flow.^20^ This knowledge is exquisitely important for the design of artificial channels^21–27^ in material science, where the selectivity and permeability mechanism of AQPs serve as template to design artificial water channels envisioned to be used in next generation membrane-based separations and purifications. Similarly, AQPs^28–35^ itself or carbon nanotubes^36^ are potential building blocks of biomimetic membranes. These highly permeable pore structures are envisioned to determine membrane performance, selectivity and functionality. To increase the stability and chemical resistance of such biomimetic membranes, lipid bilayers are replaced by polymer layers for industrial applications.^37^ Furthermore, other MPs are embedded into polymer-based membranes in the emerging field of synthetic biology.^38–40^

To understand the impact of different factors such as point mutations, post-translational modifications, external stimuli like protons or divalent ions, and lipid interactions on water permeability of MPs embedded in lipid based membranes and further advance the understanding of the molecular determinants of water flux through narrow MPs it is necessary to assess single channel permeability (*p*_f_) values with highest accuracy. Otherwise, relative changes in MP activity in dependence of *e.g.* the lipid surrounding or the oligomeric state could be dismissed as measurement artefacts. Likewise, the performance of artificial and biological channels in biomimetic membranes, as well as MP stability and functionality in polymer-based membranes can only be assessed by calculating accurate *p*_f_ values. Estimation of *p*_f_ values involves the precise determination of (i) the overall (membrane + channels) membrane water permeability *P*_f_ and (ii) the background (lipid or polymer matrix) permeability *P*_m_ as well as (iii) channel (MP or artificial channels) counting as1
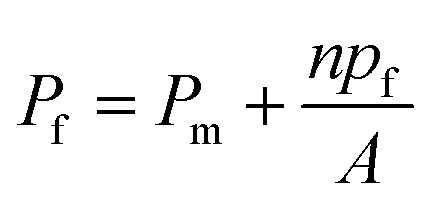
with *n* being the average number of monomers (smallest water transporting units) per vesicle and *A* the area of the lipid or polymer membrane.

**Fig. 1:**
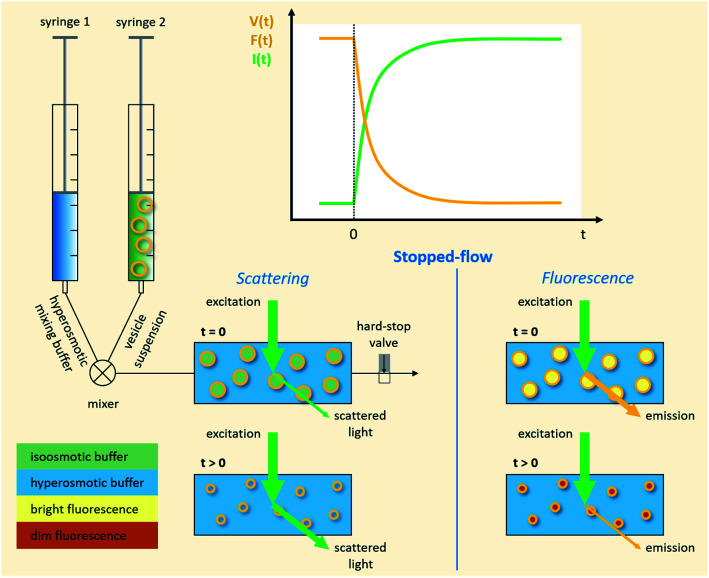
Schematic representation of a stopped-flow device. Two syringes containing the vesicle suspension and hyperosmotic mixing buffer, respectively, are driven by independent stepping-motors. The content of the two syringes is rapidly mixed at the mixer and transferred into the observation chamber (cuvette). The time it takes for the solution to flow from the mixer, where the reaction starts, to the cuvette is called ‘dead time’ and is usually in the low millisecond range. A hard-stop valve abruptly stops the liquid flow, which eliminates pressure artifacts. Monochromatic light of defined wavelength illuminates the chamber and at a detection angle of 90° either the scattered light, or by introducing a longpass filter only the emitted fluorescent light is detected by a photomultiplier.

Different methods exist to estimate such *P*_f_ and *P*_m_ values.^18,41,42^ The most widely used method involves the rapid exposure of large unilamellar vesicles (LUVs) or polymersomes (LPSs) with a diameter of around 100–150 nm to an osmotic gradient by a stopped flow device (Fig. 1). Such lipid^43,44^ and polymer^45–47^ vesicles can be directly imaged using transmission electron microscopy. From these studies it is evident that vesicle extrusion leads to a distribution of vesicle sizes which are rather spherical under isosmotic conditions.^43^ Thereby, the homogeneity and unilamellarity of a vesicle suspension increases using multiple extrusion cycles and smaller (*e.g.* 100 nm) pore sizes.^43^ Furthermore, their shape transforms upon application of a hyperosmotic gradient due to the increased surface to volume ratio.^44–47^ However, due to their small size, no method exists to directly assess the dynamically changing vesicle volume as for example by light microscopy in the case of giant unilamellar vesicles (GUVs).^48^ Instead the resulting change in vesicle volume can either be tracked by detecting changes in intensity of scattering light^49^ or fluorescence.^50^ In both cases, the change in vesicle volume upon application of a hyperosmotic gradient can be written as2
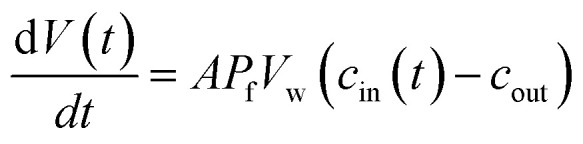
where *V*(*t*), *A*, *P*_f_, *V*_w_, and *c*_out_ are the vesicle volume at time *t*, the surface of the vesicle, the water permeability of the vesicular membrane, the molar volume of water and the osmotic concentration of the osmolyte in the external solution. The osmotic concentration *c*_i_ (also known as osmolarity) of substance *i* is related to the molar concentration *c̃*_*i*_ by:

3 *c*_*i*_ = *φ*_*i*_*n*_*i*_*c̃*_*i*_

where *φ*_*i*_ and *n*_*i*_ are the osmotic coefficient and the number of particles into which molecule *i* dissociates.

**Fig. 2:**
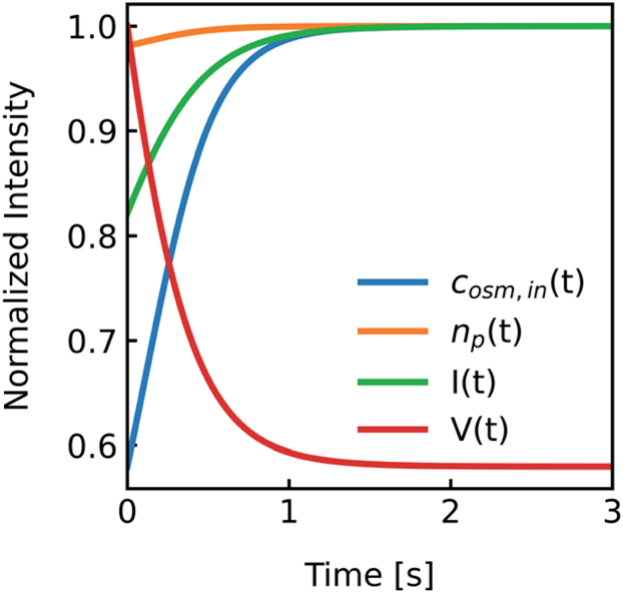
Normalized measurement parameters after subjection to a hyperosmotic solution. Plot of total osmotic concentration inside the vesicle *c*_osm,in_, refractive index of the particle *n*_p_, scattering intensity *I*(*t*), and volume *V*(*t*) over time. The starting values are: *c*_osm,in_(0) = 197 mOsm L^−1^, *n*_p_(0) = 1.335, *I*(0) = 1.3 × 10^−5^ AU, *V*(0) = 2 × 10^−13^ μL. For all simulations in this paper, except explicitly mentioned, typical experimental conditions have been used: 187 mOsm L^−1^ NaCl and 10 mOsm L^−1^ buffer inside and outside the vesicles, additional 150 mOsm L^−1^ sucrose outside for hyperosmotic shrinkage, *P*_f_ = 6 μm s^−1^, membrane thickness *d* = 4 nm, wavelength of the illuminating monochromatic light WL = 546 nm and Weibull distribution parameters for number distribution *α* = 1.35, *β* = 12.1 and *μ* = 32.6 with expected values for the radius of ∼44 nm for number, 50 nm for volume and 57 nm for intensity weighted distributions (see the corresponding curves in Fig. 3A).

In case the permeation of other substances is negligible compared to water permeation the time dependent osmotic concentration of the osmolyte inside the vesicle *c*_in_(*t*) can be expressed as4
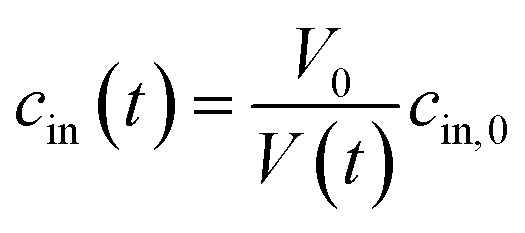
with *V*_0_ and *c*_in,0_ the initial vesicle volume and the initial osmotic concentration of the osmolyte inside the vesicle. Assuming a solute is membrane impermeable at the timescale of the experiment, internal solutes are up concentrated until the osmolarity of the internal and the external solutions match, causing a refractive index change of the vesicle during shrinkage. Whereas fluorescence self-quenching is a direct read out of the vesicle volume, the change in the scattering signal *I*(*t*) is influenced by the change in vesicle volume and the refractive index of the vesicle (Fig. 2).

To accurately fit the volume change after subjection to a hyperosmotic solution and calculate experimentally derived *P*_f_ values it is possible to use either the analytical solution^19^ to eqn (2)5
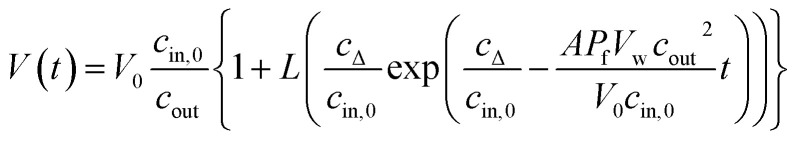
where *c*_Δ_ = *c*_out_ − *c*_in,0_, or a suitable approximation^18^6
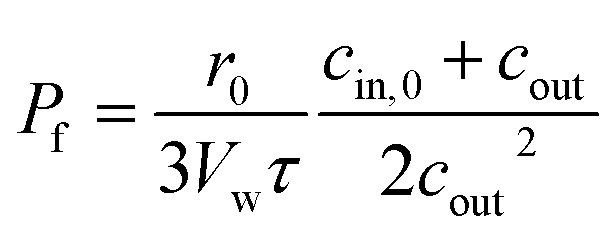
with *r*_0_ and *τ* the initial average radius of the vesicle population and the time constant of an exponential fit to the data. However, besides choosing correct models instead of historical but erroneous approximations, which may lead to *P*_f_ values being orders of magnitude off,^18^ it is also important to consider several other methodological peculiarities of scattering and self-quenching experiments on LUVs and LPSs. Herein, it is our intention to thoroughly discuss and review all these points necessary to contemplate when performing *P*_f_ measurements with a stopped-flow device in general or using scattering or self-quenching experiments in particular. This section will be followed by an example of residual detergent after MP reconstitution illustrating the strengths and weaknesses of both approaches.

## 2. Material and methods

### 2.1. Protein overexpression and purification of AQP1

*S. cerevisiae* strain pep4 was transformed with expression vector pYES2-His-YFP-AQP1 encoding human wildtype AQP1 N-terminally fused to a 10 times His-Tag and YFP. After clonal expansion in 500 mL DOB-Ura till O.D.600 was 1.0, protein expression was induced by transferring the cells to 3l YPG medium for 16 hours. Harvested cells were resuspended in one 1/100 culture volume of ice-cold lysis buffer (100 mM K_2_HPO_4_, protease inhibitors, pH 8.0) and subjected to three lysis cycles using EmulsiFlex (Avestin) at 20 000–25 000 psi (4 °C). Unbroken cells and debris were separated from the cell lysate by a 20 min centrifugation at 7000 × *g* and discarded. Membrane fractions recovered from the supernatant by a 120 min centrifugation at 100 000 × *g* were resuspended to the original volume in solubilization buffer [3% OG in 100 mM K_2_HPO_4_, 10% (vol/vol) glycerol, 200 mM NaCl, pH 8.0] and incubated for 1 hour at 4 °C on a roller shaker. Insoluble material was pelleted by 60 min centrifugation at 100 000 × *g*. The soluble fraction was mixed with 2 mL of prewashed Ni-NTA-agarose beads (Qiagen) and incubated with agitation at 4 °C for 60 min. The beads were then packed in a plastic disposable column (Stratagene) and washed with 100 bead volumes of wash buffer (3% OG, 100 mM K_2_HPO_4_, 10% glycerol, 200 mM NaCl, 100 mM imidazole, pH 7.5) to remove nonspecifically bound material. Ni-NTA-agarose-bound material was eluted 5 times sequentially by adding 0.5 mL elution buffer (3% OG, 100 mM K_2_HPO_4_, 10% glycerol, 200 mM NaCl, 0.5 M imidazole, pH 7.5) at each step. Typically, eluates of the 3 l expression cultures yielded pure protein at a concentration of 1.5 mg mL^−1^, measured by Bradford using BSA as a standard.

### 2.2. Reconstitution

*E. coli* polar lipids (PLE, Avanti Polar Lipids, Alabaster, AL, USA) was dissolved in chloroform, labeled with Atto633PPE and dried on a rotary evaporator. The dry lipid film was rehydrated with reconstitution buffer (100 mM NaCl, 10 mM MOPS, 1.33% OG, pH 7.4) to a final lipid concentration of 20 mg mL^−1^. After 10–15 minutes of bath sonication, the vesicle suspension was mixed with the purified protein and incubated for 1 h at room temperature. With increasing amount of Bio-Beads SM-2 resin (Bio-Rad Laboratories, Hercules, CA, USA) detergent was removed in 3 steps at 4 °C within 48 h. Proteoliposomes (PL) were pelleted by 100 min centrifugation at 100 000 × *g*, resuspended in reconstitution buffer without OG and finally extruded through two polycarbonate filters with 100 nm pore sizes.

### 2.3. Bare lipid vesicle preparation

Large unilamellar vesicles (LUVs) were prepared from an *E. coli* polar lipid extract (PLE) in chloroform as previously described.^51^ In brief, PLE was dried on a rotary evaporator and hydrated in working buffer (100 mM NaCl, 10 mM MOPS, pH 7.4). Subsequently, the solution was extruded through 100 nm polycarbonate filters to reach a final stock solution of 10 mg mL^−1^. For self-quenching experiments with carboxyfluorescein (CF), if not differently stated, 10 mM CF were added during lipid rehydration. Directly before the measurements free dye was removed *via* PD-10 columns.

### 2.4. Vesicle size distribution & mean diameters

The size distribution of vesicles formed by extrusion or detergent removal is well described by the Weibull distribution.^52^ Thereby, the probability density function (pdf) of the general Weibull distribution is given by7
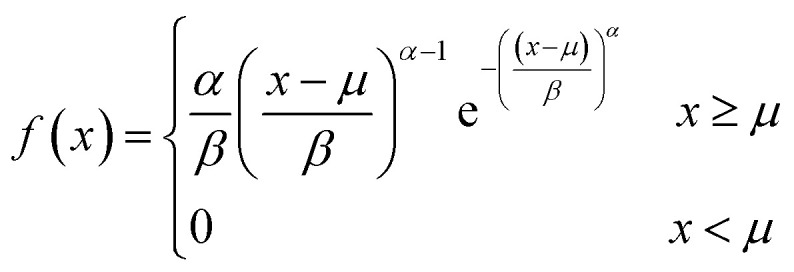
where *α*, *β* and *μ* are the shape, scale and location parameter (Fig. S1†). The corresponding cumulative density function is:8
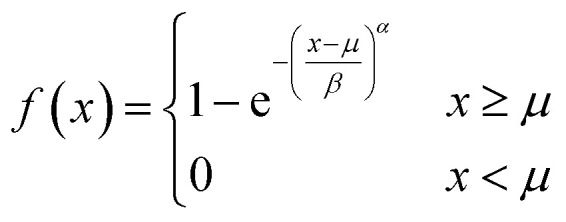


**Fig. 3:**
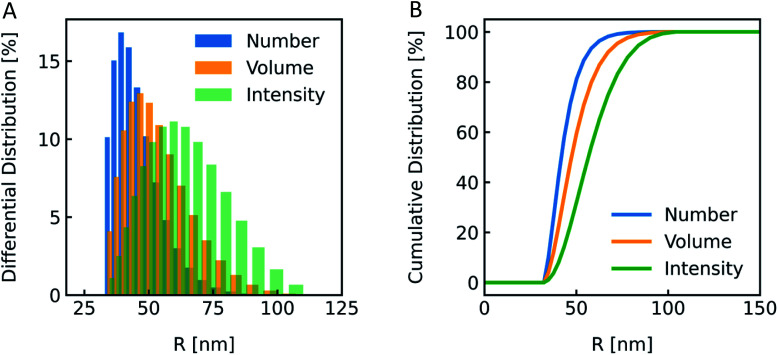
Size distribution analysis by dynamic light scattering (DLS). (A) Number (blue), volume (orange) and intensity (green) weighted distributions representing the number of vesicles in % with radius *R* and (B) the corresponding cumulative distributions. PLE LUVs were measured with DLS after extrusion through a 100 nm filter.

The most straightforward method to measure such vesicle size distributions is dynamic light scattering (DLS). We used a DelsaNano HC particle analyzer (Beckman Coulter; Brea, CA, USA) to measure the scattering distribution (green bars and line in Fig. 3) at RT and at a fixed angle of 165° and recalculated it to a volume (orange) and a number distribution (blue) with the supplied software. Notably, the RGD theory shows a blind spot for lipid vesicles with a diameter of approximately 250 nm at a detection angle of 165° (Fig. S2†). According to the Rayleigh approximation, the intensity distribution is proportional to *r*^6^ and the volume distribution to *r*^3^. Thus, the intensity weighted distribution can be converted into a volume weighted distribution with9
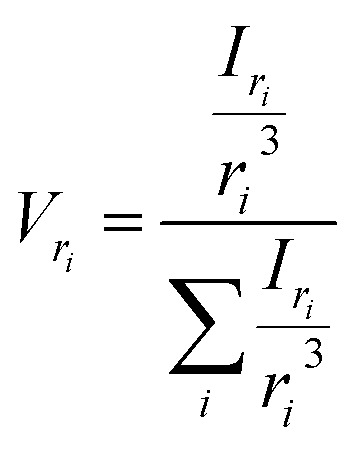
where *I*_*r*_*i*__ and *V*_*r*_*i*__ are the relative amount of scattered intensity of particles with size *r*_*i*_ and the volume-weighted distribution for particles with radius *r*_*i*_, based on the volume of particles with size *r*_*i*_.^53^ The corresponding normalized number-weighted distribution *N*_*r*_*i*__ for particles with radius *r*_*i*_, based on the number of particles with size *r*_*i*_ is therefore10
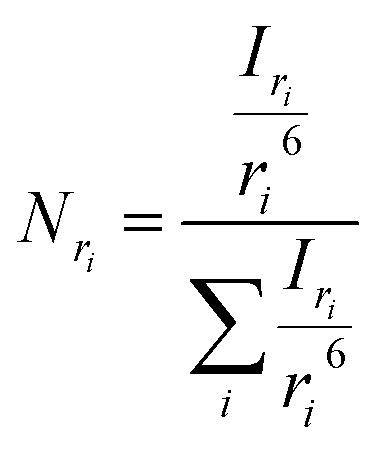


The vesicle radius is derived from the peak of the volume (for self-quenching experiments)^33^ and the cumulant radius of the intensity (for scattering experiments) distribution.

Generally, volume distributions can be compared to the results of fluorescence correlation spectroscopy (FCS)^54,55^ whereas size distributions obtained with electron microscopy (EM)^56^ are closest related to the number distributions. For the transformation of intensity to volume and number weighted distributions, it is assumed that particles are spherical and homogeneous,^57^ the optical properties are known (*e.g.* refractive indices), and that the determined intensity distribution is correct. Hence, due to the assumptions inherent in the transformation, the results of volume and number distribution always need to be taken with caution.

### 2.5. Fluorescence correlation spectroscopy (FCS)

FCS served to measure the average radius of vesicle ensembles and the leakage of CF from lipid vesicles after subjection to an hypoosmotic gradient. In brief, the average residence time *τ*_D_ of Atto633-PPE labeled vesicles and the appearance of free CF visible as a second component in the autocorrelation function *G*(*τ*) of the fluorescence temporal signals from the confocal volume, was acquired using a commercial laser scanning microscope equipped with avalanche diodes (LSM 510 META Confocor 3 with a 40x-UOPLAN water immersion objective; Carl Zeiss). The confocal volume was calibrated using the residence time of rhodamine 6G in solution and its diffusion coefficient of 426 μm^2^ s^−1^,^58^ as previously described.^59,60^ To this end, we applied the standard model for one- or two-component free 3D diffusion.^51,61^*D* was determined as *ω*^2^/4*τ*_D_.

### 2.6. Stopped-flow experiments & data analysis

PLs and LUVs are subjected to a hyperosmotic solution in a stopped-flow apparatus (SFM-300 or μ-SFM, Bio-Logic, Claix, France) at 4 °C and the intensity of scattered light is monitored at 90° at a wavelength of 546 nm if not otherwise stated.^1,18–20^ Water permeability values *P*_f_ are, except explicitly mentioned, calculated using our recently calculated analytical solution.^19^ For self-quenching experiments, monochromatic light of 480 nm wavelength is used to illuminate the sample. The emitted fluorescent light passes a 515 nm longpass filter and is detected at an angle of 90°. Averaged self-quenching curves are then fitted, according to eqn (5), to the following function:11
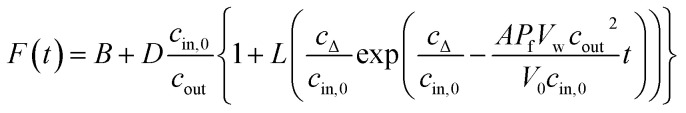
where *F*(*t*) is the fluorescence intensity and *B* and *D* are fitting parameters.

The normalized intensity *I*_norm_ is determined either by *I*_norm_(*t*) = *I*(*t*)/*I*_max_ or, for normalization between [0,1], by *I*_norm_(*t*) = (*I*(*t*) − *I*_min_)/(*I*_max_ − *I*_min_), where *I*(*t*) is the measured intensity at time *t* and *I*_max_ and *I*_min_ are the averaged maximum and minimum intensities. In all experiments, buffer osmolarities were determined with a Wescor 5500 Vapor Pressure Osmometer.

### 2.7. *I*(*t*) and *V*(*t*) simulations

For simulating the scattering behavior of size distributed vesicles, we used the number distribution determined by dynamic light scattering and fitted the resulting cumulative function by the cumulative density function of the Weibull distribution (eqn (8)). The determined Weibull parameters were then used to weight *I*(*r*,*t*) with the Weibull probability density function (eqn (7)) of *r*. Subsequent integration over *r* ranging from 1 nm to 1000 nm results in the total scattering intensity signal of the vesicle suspension. The time dependent volume change after applying a hyperosmotic gradient is simulated according to eqn (2). The relationship between vesicle volume *V*(*t*) and scattering intensity *I*(*t*) can be described by the Rayleigh–Gans–Debye relation:^19^12
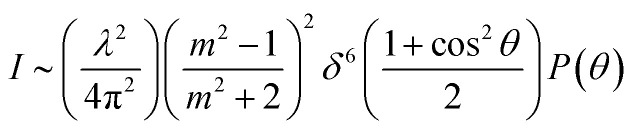
where *λ*, *θ*, *m*, *δ*, *P*(*θ*) are the effective wavelength (*i.e.* the ratio of *λ*_0_ (=546 nm in our experiments) and the refractive index *n*_s_ of the surrounding aqueous solution, the angle (here 90°) at which the intensity *I* of scattered light was measured, the relative refractive index (*m* = *n*_p_/*n*_s_, where *n*_p_ is the average refractive index of the particle), the size parameter, and the form-factor, respectively. *δ* is defined as *δ* = 2π*R*/*λ*, where *R* is the vesicle radius. The form factor for optically homogeneous vesicles (homogeneous sphere model) may be expressed *via P*(*θ*) = (3(sin *u* − *u* cos *u*)/*u*^3^)^2^ whereas *P*(*θ*) used for the hollow sphere model (see Fig. S3†) is 
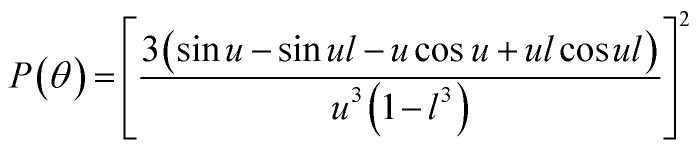
.^62,63^ In both cases, *u* = 2*δ* sin(*θ*/2). Throughout this paper, except explicitly mentioned, we used the homogeneous sphere model for simulations and fits.

The average refractive index *n*_p_ of a particle can be expressed, according to the sum rule of polarizability, from the following equation^63^13
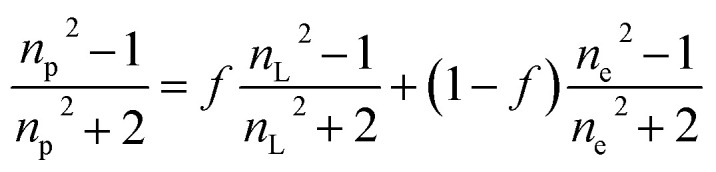
where *n*_L_, *n*_e_ and *f* are the refractive indices of the lipid (*n*_L_ = 1.497)^62^ and the intravesicular solution and the fraction of the lipids per vesicle, which can be expressed as a function of *R*_0_, *R*, and *h*, the initial vesicle radius, the radius of the osmotically shrunken vesicles and the thickness of the lipid bilayer as14
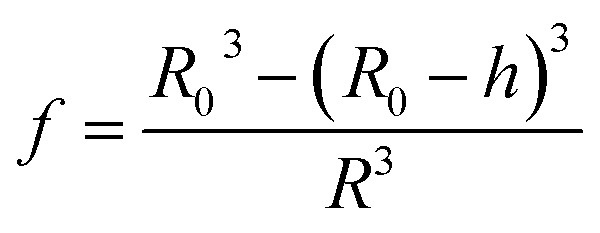


Thus, with eqn (13), we obtain for the average refractive index of a particle15
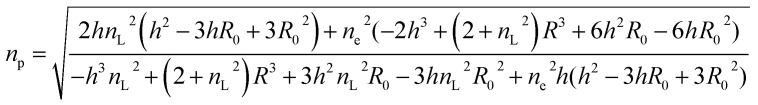


**Fig. 4:**
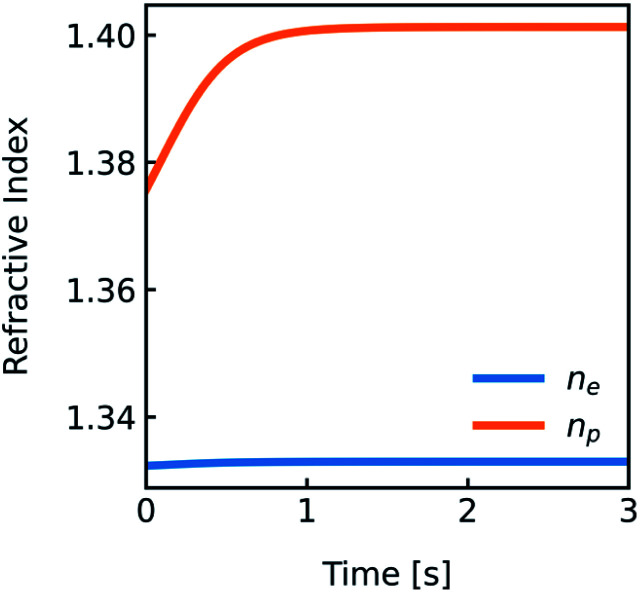
Development of the refractive indices *n*_e_ and *n*_p_ over time. Simulation conditions are described in Fig. 2.

Vesicle shrinkage leads to an increase in internal electrolyte concentration (Fig. 4):16
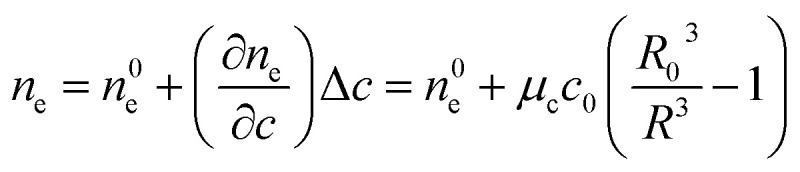
where *n*^0^_e_, *μ*_c_ and *c*_0_ are the initial refractive index, the concentration coefficient for the refractive index and the initial solute concentration.

The temperature dependence of the refractive indices *n*_e_ and *n*_s_ is taking into account with:17
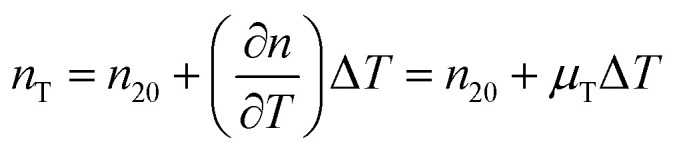
where *n*_T_, *n*_20_ and *μ*_T_ = −1.2 × 10^−4^ K^−1^ ^64^ are the refractive index at temperature *T*, the refractive index at 20 °C and the temperature coefficient for the refractive indices, respectively.

## 3. Results

It is our aim to extensively examine various methodological aspects of water permeability estimation using stopped-flow spectroscopy. This should on the one hand foster the understanding of methodological peculiarities and on the other hand serve as a reference study regarding the choice and interpretation of measurement conditions and data. Therefore, we first consider scattering and self-quenching relevant details before we for the first time thoroughly assess the effect of vesicles-size-distributions, MP-distributions between PLs and the choice of osmotic measurement conditions on *P*_f_. Finally, we use a showcase experiment demonstrating the effect of rest detergent in lipid vesicles after MP reconstitution on the scattering and self-quenching approach to measure *P*_f_ values with stopped-flow.

### 3.1. Scattering

#### 3.1.1. Relation: *I*(*t*)–*V*(*t*)

**Fig. 5:**
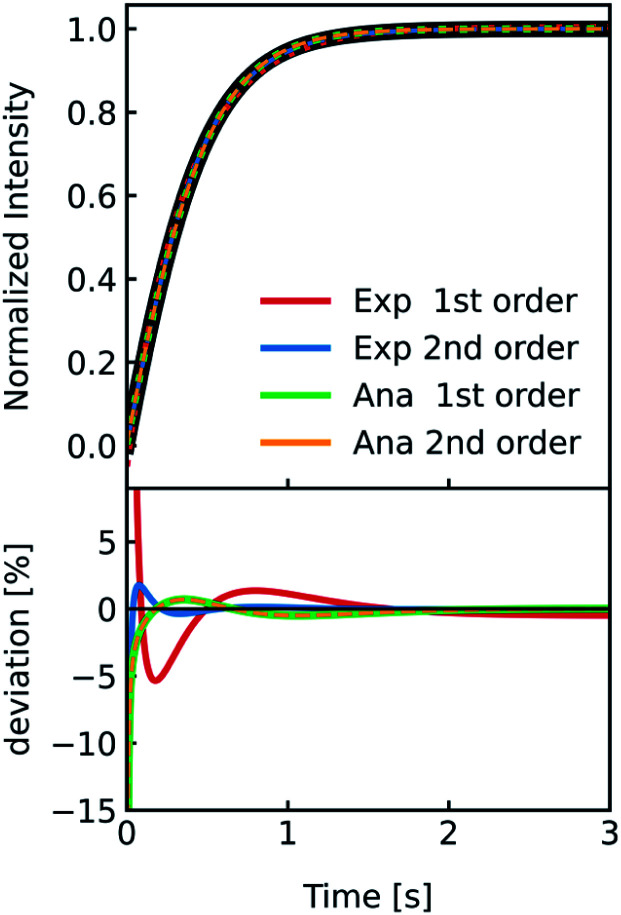
Comparison of methods relating *V*(*t*) and *I*(*t*). Stopped-flow simulation of osmotic shrinkage of liposomes with Weibull distributed radii and a water permeability *P*_f_ = 6.0 μm s^−1^ (black curve). For the simulation, standard conditions described in Fig. 2 have been used. The simulated scattering curve was fitted with an exponential using a *I*(*t*)–*V*(*t*) relationship described by a 1^st^ (red) and 2^nd^ order (blue) Taylor approximation and with the analytical solution using again the 1^st^ (green) and 2^nd^ order (orange) Taylor approximation. The deviation of the different fitting routines to the simulated scattering curve is depicted below. With the right choice of constraints concerning the signs of the fit parameters (negative *b* and positive *d* in eqn (18)), the implementation of the 2^nd^ order Taylor approximation leads to virtually the same results as with using the 1^st^ order Taylor approximation for fitting with the analytical solution, whereas for the exponential fit, the 1^st^ order Taylor approximation is the best choice (see the corresponding *P*_f_ values in table S1†).

As comprehensively explained in the Material and methods section “*I(t) and V(t) simulations*” light scattering at lipid vesicles with a size well below the optical resolution limit can be described by the RGD theory. However, these scattering intensities *I*(*t*) are only an indirect measure of vesicle volume *V*(*t*). Hence, for *P*_f_ calculations it is critical to relate *V*(*t*) to *I*(*t*). In the past this was done using a second-degree polynomial function^19^ and empirical approximations ranging from double logarithmic,^65^ over quadratic^66^ to simple linear relations.^67^ As this point of signal translation is always a point of confusion, we directly compare the differences in *P*_f_ for a second-degree polynomial function,

18 *I*(*t*) = *a* + *bV*(*t*) + *dV*^2^(*t*)

which should be the most accurate approximation as scattered light intensities exhibit an inflection point on *R*,^19^ with a linear relation,

19 *I*(*t*) = *a* + *bV*(*t*)

which is the simplest approximation. The latter is commonly used together with exponential fits to the scattering data. To be able to attribute differences of both approaches on *P*_f_ we simulated *I*(*t*) for a common vesicle ensemble, related it to *V*(*t*) by both approaches and then fitted *V*(*t*) with the analytical solution (eqn (5)) and an exponential fit. Further, we used the time constant *τ* of the exponential fit to calculate *P*_f_*via* the most accurate approximation depicted in eqn (6). Fig. 5 illustrates that all four approaches nicely fit the simulated data with only minor deviations (<10%). Solely, estimation of *P*_f_ with an exponential fit using a 2^nd^ order Taylor approximation leads to an error of 30% in our case (Table S1†).

#### 3.1.2. Excitation wavelength

**Fig. 6:**
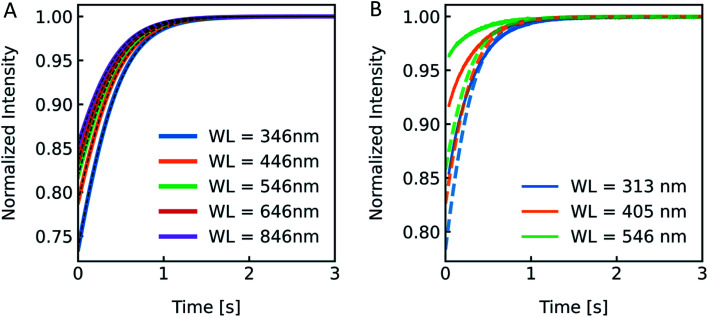
Excitation wavelength influences *I*(*t*). (A) Normalized scattered light intensity upon exposure to a hyperosmotic gradient for different wavelengths WL of the illuminating monochromatic light. (B) Simulated (dashed lines) and experimental scattering data (solid lines) for different wavelengths.

According to the RGD relation (eqn (12)) scattering intensities depend on the experimentally used excitation wavelength. Furthermore, a variety of excitation wavelengths in the range of 450 nm,^68^ 500 nm,^69^ 546 nm ^19^ to 600 nm ^33^ were exploited for scattering experiments in literature. To visualize the effect of *λ*_ex_ on the experiment and to guide the rational selection of *λ*_ex_, we simulated and measured *I*(*t*) at different *λ*_ex_. From eqn (12) the relation of *I*(*t*) on *λ*_ex_ is not directly obvious as *λ*_ex_ is not only hidden in *δ* but also in *P*(*θ*). Generally, the RGD theory is applicable in the visible light range if the conditions |*m* − 1| ≪ 1 and *kd*|*m* − 1| ≪ 1 with *k* = 2π/*λ* and *d* the vesicle diameter are fulfilled.^70^ Simulation of scattering data at different wavelengths ranging from 346 nm to 846 nm reveal an increasing relative signal amplitude with smaller wavelength (Fig. 6A). Next, we chose the three main intensity peaks in our XeHg light source to verify that a similar dependence can also be seen experimentally (Fig. 6B). The discrepancies in the relative signal amplitudes between *in vitro* and *in silico* data may arise from the wavelength dependence of the refractive indices, which have not been taken into account with our simulations. Another source of error quantifying the relative amplitude change of experimental data is the background correction, where the scattering intensity signal of the background, which is determined by measuring the scattered light intensity of the mixture of buffer without vesicles and osmolyte buffer, is subtracted before normalization. Small errors in the determination of the background signal may have, due to normalization, a high impact on the relative amplitude change. Nevertheless, the kinetic and thus the *P*_f_ values are not affected by the height of the relative amplitude. Fitting the data with the analytical solution illustrates that *P*_f_ is within the error of the measurement as expected and with these experimental conditions *P*_f_ is independent of the wavelength used (Fig. S4 and Table S2†).

#### 3.1.3. Choice of osmolyte

**Fig. 7:**
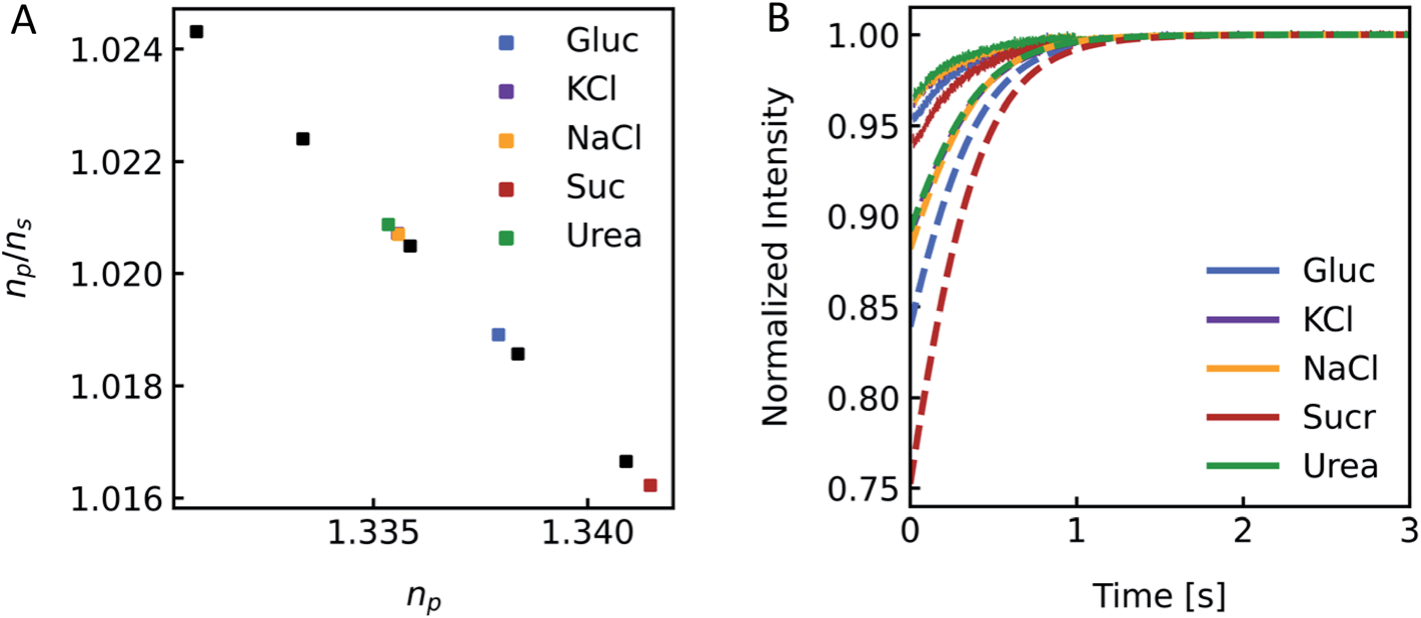
Impact of the osmolyte on the refractive index and *I*(*t*). (A) Ratio of the refractive indices of vesicles *n*_p_ to bulk buffer *n*_s_, with 100 mM NaCl in – and outside and additional 150 mM of glucose (blue), potassium chloride (purple), sodium chloride (orange), sucrose (red) and urea (green) outside of the vesicles. (B) Simulated (dashed lines) and experimental (solid lines) scattering traces for the different osmolytes using standard conditions (see Fig. 2).

Different osmolytes are being exploited for measuring water permeabilities with stopped-flow. Common osmolytes include sucrose, urea, glucose and various salts to name a few. The most obvious impact of the osmolyte on the experiment is the permeability of (i) the lipid or polymer matrix or (ii) the protein itself to the osmolyte of choice. While in the first case the osmotic gradient diminishes with time, which effects *P*_f_ only indirectly, in the latter case, the osmolyte also directly impacts the water flux through the channel. Depending on the ratio of solute permeability *P*_s_ to *P*_f_, the solute blocks or slows down the passage of water through the channel. This slow down or inhibition of *P*_f_ was shown for example for K^+^ in the bacterial potassium channel KcsA^2^ and glucose in the human sodium glucose cotransporter hSGLT1.^1^ However, this effect can also be exploited to measure the equilibrium dissociation constant of the osmolyte to the respective channel.^2^ In addition, the osmolyte influences scattering measurements due to their sensitivity to the refractive index of the particle. This increase in inner osmolarity during vesicle shrinkage increases the vesicle's refractive index, further elevating their light scattering ability. At the same time, the reduced vesicle size, counteracts this effect by diminishing its scattering ability. Fig. 7 illustrates the influence of the refractive index of the osmolyte on *I*(*t*). To increase the relative signal amplitude, it is necessary to minimize the refractive index of the vesicle *n*_p_ which minimizes the refractive index difference between the vesicle and the surrounding *n*_p_/*n*_s_.

#### 3.1.4. Membrane thickness

**Fig. 8:**
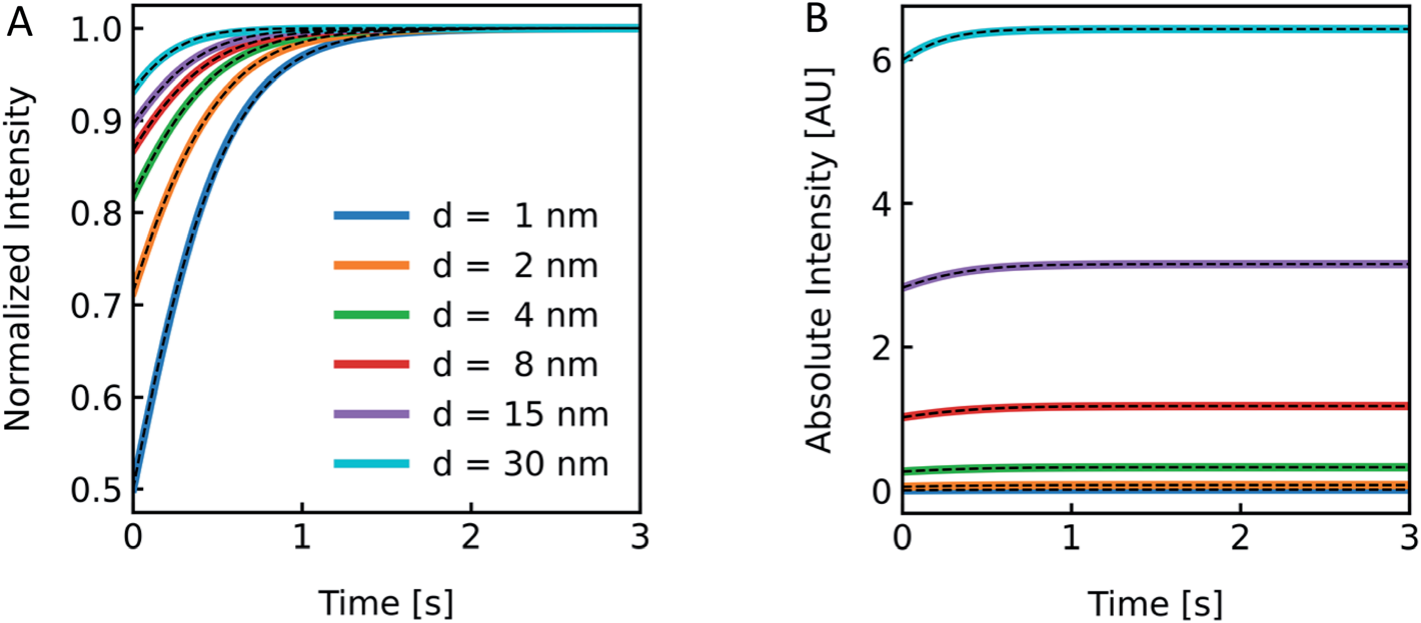
Effect of membrane thickness on *I*(*t*). Normalized (A) and absolute (B) scattered light intensity of lipid vesicles upon exposure to a hyperosmotic gradient for different values of membrane thickness ranging from 1 nm (blue), 2 nm (orange), 4 nm (green), 8 nm (red), 15 nm (purple) to 30 nm (cyan). The corresponding *P*_f_ values from analytical fits to the simulated data (black dashed lines) are depicted in Table S3.† As we are only interested in qualitative comparison between different membrane thicknesses, we used a similar refractive index for the refractive index of the polymer as compared to the lipid throughout the manuscript.

Polymer membranes forming LPSs may be several times thicker than lipid membranes. Whereas lipid bilayers of biological membranes exhibit a membrane thickness *d* in the range of 3–4 nm,^41,71^ polymer membranes range from 5–21 nm.^72,73^ To illustrate the effect of such an increased *d* on *I*(*t*) we simulated scattering intensities for a variety of *d* values. As it can be seen in Fig. 8, the relative amplitudes increase with lower *d* values, but with the absolute values of *I*(*t*) increasing at thicker membranes. This can be rationalized by the fact that the lipid/polymer contribution to *n*_particle_ always exceeds that of the inner vesicle solution. As summarized in Table S3,†*P*_f_ and its deviation from the simulated values increase with increasing *d*.

#### 3.1.5. Vesicle shape

Both, the form factor *P*(*θ*) used in the RGD theory as well as the change in vesicle volume upon application of a hyperosmotic gradient (eqn (2) and (4)) assume a spherical vesicle shape. However, vesicles prepared by extrusion can exhibit shapes, such as prolate and oblate ellipsoids^74^ and subjection to a hyperosmotic gradient leads to a volume decrease of the vesicles by a factor of *c*_out_/*c*_in,0_. Hence, as the surface area of a lipid vesicle is assumed to stay invariant during shrinkage, vesicle shape deviates more and more from a spherical shape during shrinkage at larger hyperosmotic gradients. Still, *P*_f_ was found to be invariant in scattering experiments under hyperosmotic conditions.^18^ Furthermore, we simulated scattering signals for hyperosmotic shrinkage with increasing ellipticity (Fig. S5†) according to the form factor for prolate and oblate vesicles.^74^ These simulations clearly showed that despite an increased absolute scattering intensity at higher ellipticity the effect of vesicle deformation on the overall shape of the scattering trace (Fig. S5A†) and *P*_f_ can be neglected. Therefore, we can conclude that the vesicle shape has a marginal influence on *I*(*t*).

### 3.2. Self-quenching

Fluorescence self-quenching experiments assume a linear relation between *V*(*t*) and the fluorescence signal *F*(*t*). Even though the absolute number of fluorophores stays constant during vesicle shrinkage, the concentration inside the vesicles increases. This leads to a decreased average distance between the fluorophores, increasing fluorescence self-quenching and therefore reducing the overall signal intensity. Generally, *F*(*t*) of a single fluorophore due to self-quenching for a given concentration is described by the Stern–Volmer relationship20
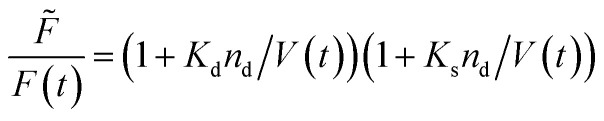
where *F̃*, *K*_d_, *K*_s_ = 412 M^−1^,^75^*n*_d_ and *V* are the fluorescent intensity in absence of quencher, the Stern–Volmer constant for dynamic quenching, the Stern–Volmer constant for static quenching, the amount of fluorescent molecules in mole and the volume of the vesicles, respectively. As dynamic or collisional self-quenching of CF in liposomes can be neglected,^75^eqn (20) reduces to21
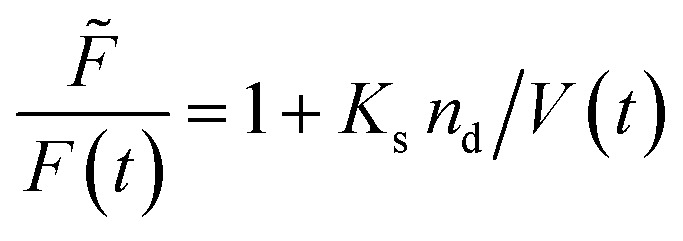


**Fig. 9:**
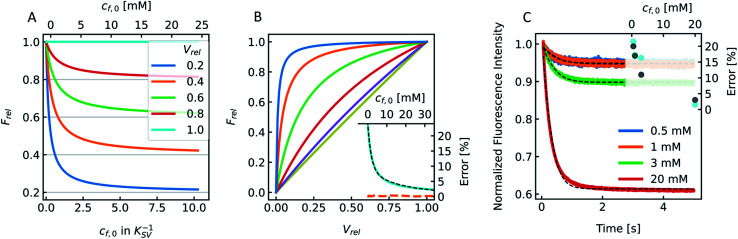
Stern–Volmer relationship. (A) Relative fluorescence of a single fluorophore *F*_rel_ over initial fluorophore concentration *c*_f,0_ in units *K*_SV_^−1^ and in mM for several values of the total relative volume change *V*_rel_ starting from 1 (cyan, no shrinkage) up to 0.2 (blue), where the final volume is 20% of the initial one. (B) *F*_rel_ plotted over *V*_rel_ for 0.025 mM (blue), 0.1 mM (orange), 0.5 mM (green), 2 mM (red), 9 mM (violet) and 40 mM (olive) initial fluorophore concentration. The error in *P*_f_ (cyan) that results from the non-linear dependence of *F*_rel_ on *V*_rel_ for low *c*_f,0_ is shown in the inset (osmotic shrinkage scattering curves depicted in Fig. S6† have been fitted with eqn (11) and the resulting *P*_f_ values have been used to calculate the error according to eqn (23)). The concentration dependent error has been fitted with a two-component exponential function (black dashed line) and the results have been used to correct the *P*_f_ values according to eqn (S2).† The deviation of the corrected *P*_f_ values to the simulated ones is shown in the inset (orange dashed line). (C) Measured hyperosmotic self-quenching curves for different concentrations of CF starting from 0.5 mM to 20 mM. The error in *P*_f_ compared to scattering measurements of the same samples is shown as cyan dots in the inset, whereas the grey dots represent the simulated values.

Thereby, the relative fluorescence signal *F*_rel_ for a given [fluorophore] is (Fig. S6†)22
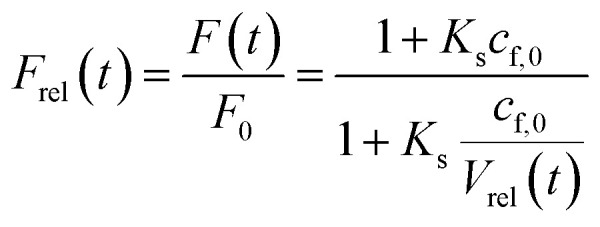
with *F*_0_, *F*(*t*), *c*_f,0_ and *V*_rel_(*t*), the initial fluorescence intensity, the fluorescence intensity at time *t*, the initial concentration of fluorescent molecules encapsulated in the vesicles and the relative volume *V*_rel_(*t*) = *V*(*t*)/*V*_0_, which is the quotient of the vesicle volume at time *t* and the initial volume, respectively. Fig. 9A visualizes this dependence of *F*_rel_ on different *c*_f,0_ for five *V*_rel_. It can be seen that *F*_rel_ approaches a stable value linearly proportional to *V*_rel_ at higher *c*_f,0_. To further illustrate that this linear relation between *V*(*t*) and *F*(*t*) does not hold for low *c*_f,0_ we plotted *F*_rel_ over *V*_rel_. Fig. 9B demonstrates that the linear relation holds for *c*_f,0_ > 10 mM with the olive curve exhibiting perfect proportionality between quenching signal and volume change. Nevertheless, smaller *c*_f,0_ can be used to estimate *P*_f_ values with errors <25% (inset Fig. 9B). A similar dependence of *P*_f_ on *c*_f,0_ was found experimentally using DPhPC (4ME 16:0 PC, 1,2-diphytanoyl-*sn-glycero*-3-phosphatidylcholine) vesicles (Fig. 9C). The error that results from the non-linear dependence for low initial fluorophore concentration has been calculated with23
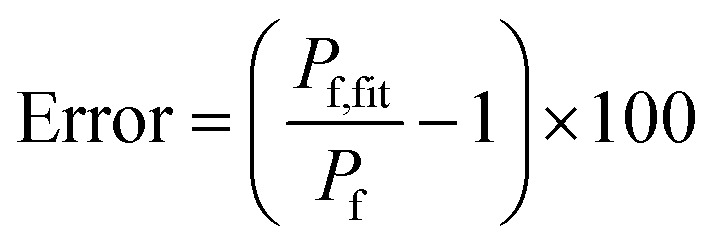
where *P*_f_ and *P*_f,fit_ are the osmotic water permeability of the simulation (shown in Fig. S6†) and the permeability value of the corresponding analytical fit.

### 3.3. General methodological peculiarities

#### 3.3.1. Hyper- or hypoosmotic conditions

**Fig. 10:**
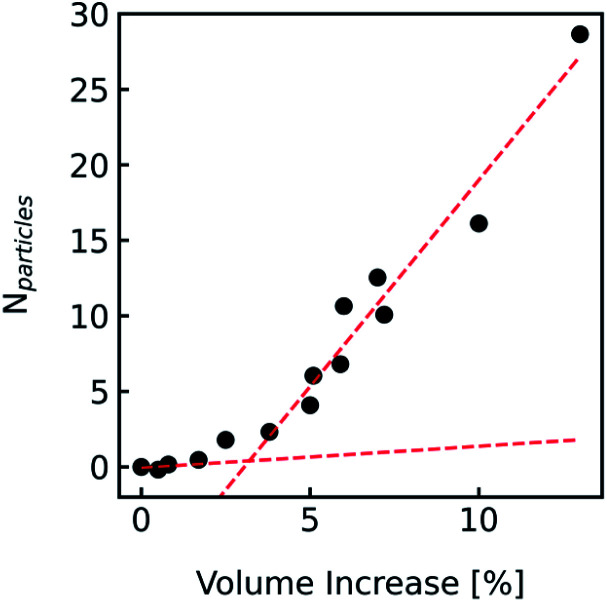
Visualization of the lost membrane integrity during vesicle swelling with FCS. Vesicles loaded with 0.5 mM CF, 100 mM NaCl, 10 mM MES, pH 7.0, were exposed to hypoosmotic buffer, which was the same buffer without addition of dye and slightly diluted with Millipore water. The measured buffer osmolarities were used to calculate the total volume change. Free dye, which leaked out due to defects in the lipid membrane during swelling, was separated from the liposomes by running a PD-10 column. With FCS the number *N*_particles_ of free CF molecules within the confocal volume was analyzed and plotted against the corresponding calculated total volume increase. Linear fits (red dashed lines) of the measured points below 2% and above 5% volume increase show a point of interception of 3.2%.

During the application of osmotic gradients to lipid- or polymer-based vesicles it is important to ensure that the integrity of the lipid or polymer membrane is guaranteed. This is the case under hyperosmotic conditions (vesicle shrinkage), but care must be taken under hypoosmotic conditions. As compared to hyperosmotic gradients, hypoosmotic gradients lead to vesicle swelling. However, lipid bilayers can only be stretched by a few percent before rupturing.^76^ From Fig. S7† it is obvious that at hypoosmotic conditions the protein concentration dependent kinetics get lost and the estimated *P*_f_ values deviate strongly from *P*_f_ values calculated from hyperosmotic data. The measured self-quenching data under hypoosmotic conditions can be explained by the outflow of fluorescent molecules during membrane rupture, which due to a decrease in [fluorophore] in the vesicle interior leads to a further reduction of fluorescent self-quenching additionally to its decrease caused by vesicle swelling. Even though the vesicle size hardly changes after application of a hypoosmotic gradient, scattering signals show a pronounced kinetic as during vesicle rupture the outflux of solute and solvent is accompanied by a change in the refractive index of the vesicle. As previously described, this decrease in *n*_p_ results in a change in *I*(*t*) like the one observed here. To corroborate our argumentation, we performed FCS experiments of CF filled LUVs which we subjected to varying hypoosmotic gradients. Fig. 10 shows quite plainly that membrane rupture takes place above a volume increase of 3.2% for PLE LUVs. Hence, vesicle rupture after subjection of LUVs to hypoosmotic conditions takes place, confirming that hypoosmotic measurement conditions cannot be chosen to estimate membrane permeability *P*_f_ without acknowledging membrane mechanics.

#### 3.3.2. Vesicle size distribution

**Fig. 11:**
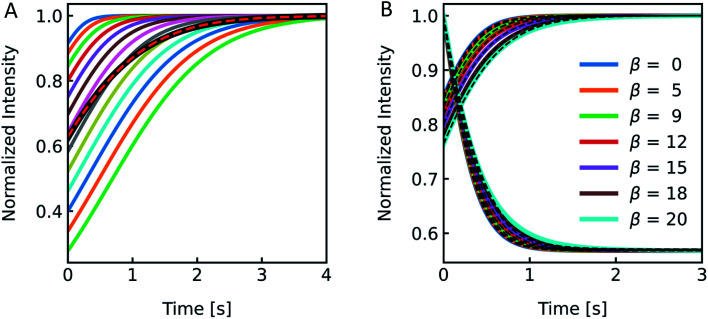
Effect of vesicle size distribution on *P*_f_. (A) Family of scattering curves and their average (black solid line) after exposure to a 150 mosm sucrose gradient. The initial equally populated radii of the vesicles were simulated in 10 nm steps ranging from 30 nm (blue) to 150 nm (green). With the intensity weighted (eqn (S1)†) average radius *R*_INT_ = 135.3 nm, the error in *P*_f_ between simulation and analytical fit was ∼5%. (B) Difference in scattering *I*(*t*) (raising curves) and self-quenching *F*(*t*) (falling curves) of largely varying scale parameters *β* of Weibull distributed vesicle size distributions. While the shape parameter *a* was set to 1.35, the location parameter γ was adjusted to achieve an expectation value for the number-weighted radius of 43.7 nm. The conversion of number to intensity distribution was done according to eqn (S3)† and the resulting distribution was fitted with a Weibull distribution function (eqn (7)) to obtain the intensity-weighted radii *R*_INT_ (see Fig. S9†). All other simulation conditions are described in Fig. 2.

The production of LUVs and LPSs or the reconstitution of MPs into PLs or LPSs never leads to a vesicle ensemble with a unique diameter. Instead, MP reconstitution and extrusion through 100 nm polycarbonate filters lead to a unimodal vesicle distribution with its width depending on the lipid composition and eventual residual detergent after reconstitution.^77^ We investigated the effect of the broadness of such a vesicle size distribution on *P*_f_ using simulated *I*(*t*) and *F*(*t*) data. First we simulated a family of scattering (Fig. S8†) and self-quenching (Fig. 11A) curves with varying *r*_0_, calculated their superimposed signal (black line) and fitted it with the analytical fit (red line). A comparison of the simulated value to the fit result calculated for the average diameter revealed an error in *P*_f_ of approx. 30% compared to an error of 4% for the self-quenching counterpart.

After this rather theoretical consideration where each individual radius had an equal probability of occurrence in the vesicle population, we simulated *I*(*t*) and *F*(*t*) (Fig. 11B) with implemented Weibull distributions (Fig. 3A). The family of curves with largely different size distributions (*β* = 0 to 20, 0 is equal to a single radius) revealed that the overall shape looks similar, but the time dependence of the respective signals seem slower (Fig. S9†) for broader distributions (larger *β*) and the relative amplitude *I*(*t*) increases as well. An analytical fit to the data (black dashed line) showed that the error in *P*_f_ for both scattering and self-quenching experiments is <12% (Fig. S9†). Thereby the chosen values in *β* cover our experimental range of AQP PLs, (poly(butadiene)–poly(ethylene oxide) (PBD–PEO)) LPSs, OSPC liposomes, PLE liposomes and PLE liposomes with detergent (OG, DM) which varied between 9 < *β* < 14.5 and 1.07 < *α* < 1.4. Similarly, the error in estimating *P*_f_ values from simulated data varying the shape parameter *α* over a reasonable range is < 18% (Fig. S10†).

#### 3.3.3. Permeability distribution

**Fig. 12:**
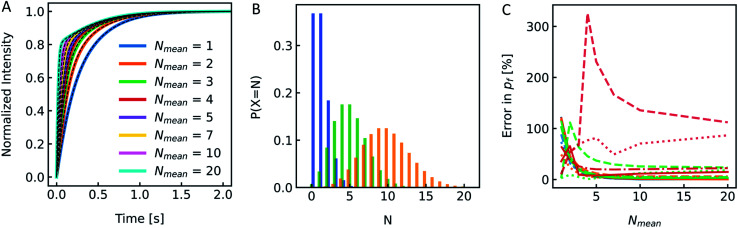
Effect of MP distribution on *P*_f_. (A) Normalized scattered light plot of osmotic vesicle shrinkage for PLs of different *N*_mean_. The fraction of vesicles which do not contain protein *x*_l_ = 0.2. For the single channel water permeability, the published value of AQP1 ^19^ with *p*_f_ = 3.25 × 10^−13^ cm^3^ s^−1^ has been taken. (B) Poisson distribution with an average number of monomers per PL *N*_mean_ of 1 (blue), 5 (green) and 10 (orange). The probability of a PL containing *N* numbers of monomers of the reconstituted protein is plotted over *N*. (C) Error in *P*_f_ depending on *N*_mean_ for *x*_l_ = 0.0 (blue), *x*_l_ = 0.2 (orange), *x*_l_ = 0.5 (green) and *x*_l_ = 0.8 (red). Dotted, dashed, dashdotted and solid lines represent the error for a two-component exponential fit with free components, a two-component exponential fit, where one component is fixed to the rate constant of liposomes containing no protein, an analytical fit and a global analytical fit, respectively. The global fit determines *P*_m_ as the average slow component (and one component for the control only) of all curves.

Reconstitution of MPs into PLs results in a Poisson distribution of the number of oligomers per PLs.^19^ Similar distributions can be assumed for artificial channels or channel forming peptides as gramicidin. To see if the distribution of *P*_f_ values in the PL fraction has a similar marginal effect on *P*_f_ as it is the case for the size distribution shown in the previous chapter, we again simulated *I*(*t*) and *F*(*t*) stopped-flow data to investigate this experimentally scarcely addressable problem. A family of simulated *I*(*t*) curves, representing a typical reconstitution series with a bare lipid vesicle fraction of 20% (*x*_l_ = 0.2) and a varying average number of MPs per PL, *N*_mean_, between 1 and 20 is shown in Fig. 12A. Three corresponding Poisson distributions (color coded) in Fig. 12B exemplify the probability of different *N*'s on the respective *N*_mean_. To estimate the effect of different *x*_l_ and *N*_mean_ on *P*_f_ we calculated the relative error in percent from the simulated *P*_f_ (Fig. 12C) by performing an analytical fit (dash-dotted lines), where the water permeability of liposomes, which do not contain channels, *P*_m_, is fixed, a global analytical fit (eqn (5)) (solid lines) or an exponential fit with two components where either one time constant is fixed (dashed lines) to the respective time constant of bare lipid vesicles or both time constants are free (dotted lines) and using eqn (6) to calculate *P*_f_. Similarly, a simulated family of *F*(*t*) curves and its error on *N*_mean_ is depicted in Fig. S11.† These simulations show that for an error in *P*_f_ below 20%, stopped-flow data should be fitted with one component fixed to the bare lipid vesicle value or a global fit, *N*_mean_ should be ≥5, and *x*_l_ ≤ 0.5. At high *x*_l_ and small *N*_mean_ the error in *P*_f_ may reach 100% or more in the worst case.

#### 3.3.4. Fitting routine – global *versus* single

The intensity signal of a vesicle ensemble after MP reconstitution subjected to a hyperosmotic gradient usually exhibits two components. As can be seen from eqn (1) the fast component *P*_f_ represents the overall water flux through the membrane of protein containing vesicles and *P*_m_ corresponding to the water transport through the fraction of empty vesicles not containing any channels. Both components must be considered by the fitting routines. As it can be seen by the example in the previous paragraph, despite the desire to optimize the reconstitution efficiency to minimize errors, the type of fitting routine is very important. It is advisable to use fitting routines were *P*_m_ can be fixed in control experiments with pure lipid vesicles or use global fits including a control sample as well as several PLs with different amounts of MPs which can be fitted by one global *P*_m_ and different *P*_f_s (global fit). We advise to use the latter as MP concentration dependent measurements ensure the highest reliability compared to results from one-point measurements.

### 3.4. Example show-case: residual detergent after membrane protein reconstitution

**Fig. 13:**
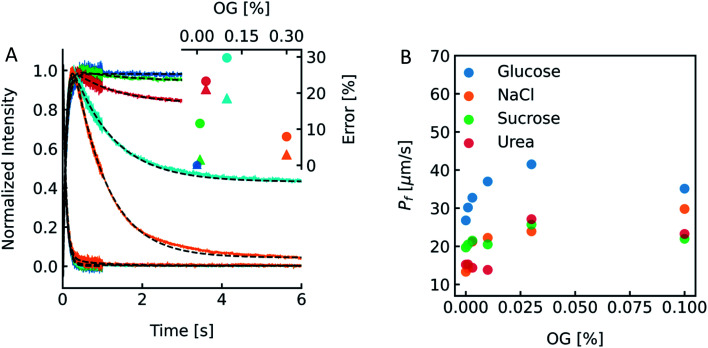
Detergent effects on scattering and self-quenching data. (A) Exemplary ensemble scattering and self-quenching traces for vesicles doped with different amounts of detergent at 24 °C. Insets: error (in %) in *P*_f_ for different detergent concentrations compared to the permeability coefficient of liposomes without detergent (dark blue) in scattering (spheres) and self-quenching (triangles) experiments. For each sample, 10 mg PLE was used with 1 mL of buffer containing 10 mM CF, 100 mM NaCl and 10 mM MOPS at pH 7.4 and the OG concentration was 0% (blue curve), 0.01% (green), 0.03% (red), 0.1% (cyan) and 0.3% (orange), respectively. (B) Dependence of membrane water permeabilities on the OG concentration. Water permeabilities were calculated from two-components exponential fits (shown in Fig. S13†).

**Fig. 14:**
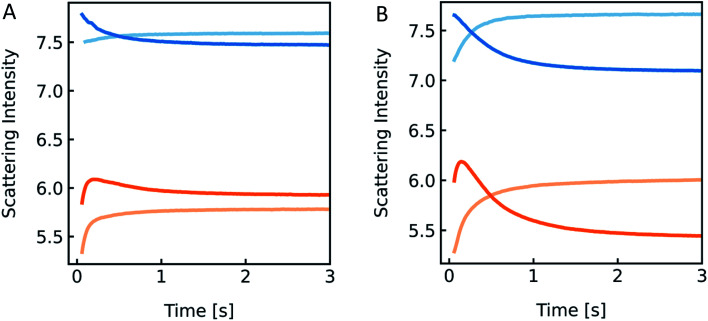
Detergent effect on scattering curves. Stopped-flow measurements of PLE liposomes (with 100 mM NaCl and 10 mM MOPS) exposed to the inside buffer (100 mM NaCl, 10 mM MOPS) (blue) and hyperosmotic buffer containing 100 mM NaCl, 10 mM MOPS and 150 mM sucrose (orange), with 0.03% OG (A) and 0.3% OG (B) only inside (dark colored) and in- and outside (light-colored) of the vesicles, respectively.

To show the differences as well as the strength and weaknesses of both approaches, we oppose them in an exemplary case face-to-face. To be able to calculate *p*_f_ values of MPs or artificial water channels, it is inevitable to reconstitute them into lipid- or polymer-based membranes. Especially in the case of MPs, this usually involves the use of detergents to mimic the lipid bilayer and avoid aggregation of MPs in solution. However, after detergent removal and PL formation it is hard to estimate if residual detergent remains in the sample and if so what the influence on the calculated *p*_f_ is. To explore a potential effect of residual detergent on *p*_f,_ we mixed different amounts of octyl-glucoside (OG) to the lipid mixture either in chloroform before evaporation (measurements shown in Fig. S13†), or in hydration buffer after evaporation (measurements shown in Fig. 13 and S12†). Note that the stated amount of detergent corresponds to the % (w/v) of OG in chloroform or in buffers used for hydration or PD-10. The final amount of detergent within the membrane is not known, as not all OG molecules have been incorporated into the lipid bilayer (see Fig. 14). After vesicle preparation the different preparations were subjected to a hyperosmotic sucrose gradient. Fig. 13A and S12† illustrate that scattering signals show a pronounced second kinetic which is elevated at higher temperatures and higher OG concentrations compared to inconspicuous *F*(*t*) data. Interestingly, even though both data sets were fitted with a two-component model to extract the first relevant time constant *τ* for *P*_f_ estimation also the seemingly unaltered *F*(*t*) data revealed a similar error in *P*_f_ (triangles) as calculations from the scattering intensity *I*(*t*) data (spheres) (Fig. 13A inset). Furthermore, scattering experiments with various osmolytes revealed similar OG concentration dependent secondary kinetics (Fig. S13†) as well as errors in *P*_f_ (Table S4,†Fig. 13B). To elucidate why the error in *P*_f_ suddenly drops further increasing the detergent concentration to 0.3% we looked into the corresponding intensity and number distribution (Fig. S14†). Both distributions illustrate that an increased OG concentration has an impact on the size distribution of the vesicles. This is evident for the sample with 0.3% OG. The number of small vesicles increases drastically and the deviation of the distribution from a Gaussian increases (red curve in Fig. S14†), which might bias the size determination and thus the corresponding results for *P*_f_.^53^

However, the perfect plateau in self-quenching experiments suggests that the reason for the second kinetic in the scattering experiments is not an OG induced enhanced solute permeability through the lipid bilayer. Alternatively, it is known that removal of OG from the lipid bilayer after subjection to detergent-free buffer is highly temperature dependent.^78^ To see if our effects were indeed due to detergent extraction from the lipid vesicles, changing the membrane area during shrinkage, we mixed lipid vesicles at two different detergent concentrations with an equal buffer or a hyperosmotic buffer, with and without detergent each (Fig. 14). Most interestingly, both samples showed a pronounced kinetic even in the isosmotic case. The decreasing signals in the dark colored curves correspond to the loss of detergent molecules upon exposure of detergent-free buffer. This effect as well as the second kinetic in hyperosmotic experiments could be reversed by the addition of detergent in the mixing solution. The concentration of OG outside the vesicles is higher for the light-colored curves, as most of the detergent inside the liposomes is supposed to be integrated in the lipid membrane. Hence, the incorporated detergent molecules increase the lipid fraction, the refractive index of the particle and therefore the intensity signal. The light orange curve represents an overlay of shrinkage and detergent incorporation, which results in the largest relative amplitude change. While the relative amplitude change within the dead time can be neglected, the final scattering intensity signal for the dark orange curve in Fig. 14B is even lower as compared to the initial signal at *t* = 0 seconds. Osmolyte influx cannot, under any circumstances, describe this effect, as the final total osmolarity after back-swelling to the initial volume would be equal to the osmolarity of the hyperosmotic buffer. The final refractive index of the particle is dependent on the final refractive indices of the lipid, which is, after back-swelling, the same as initially, and the inside solution, which is higher, due to higher concentrations. Conclusively, the final scattering intensity must be higher than the initial intensity, if there is no loss in lipid or detergent molecules.

**Fig. 15:**
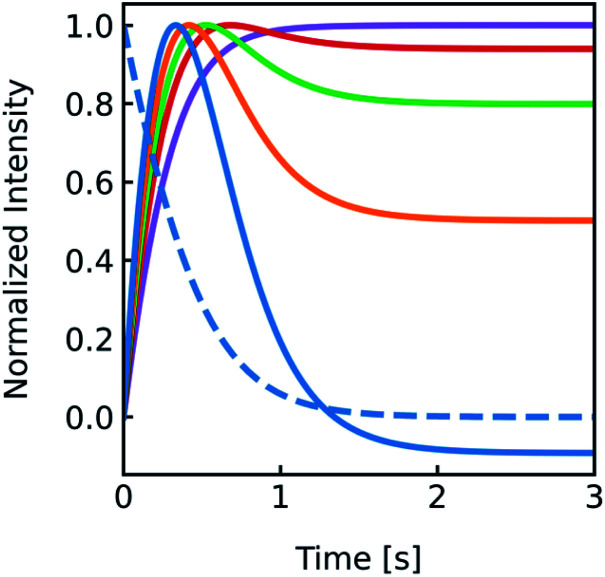
Hyperosmotic shrinkage simulations with a loss of detergent molecules. Simulations for different values of the prefactor k in eqn (24) starting from 0.20 (purple) to 0.21 (red), 0.215 (green), 0.22 (orange) and 0.225 (blue) are shown in scattering (solid lines) and self-quenching mode (dashed lines), whereas for the latter, the curves are perfectly overlaying.

To further proof that the second kinetic in the scattering traces is due to detergent extraction after subjection to a hyperosmotic detergent free solution, we simulated the effect that upon mixing OG containing vesicles with buffer lacking OG, detergent molecules leave the lipid membrane. As the influence of single detergent molecules on the total scattering intensity signal is negligible, we only take the reduction of the lipid (and detergent) fraction into consideration:24
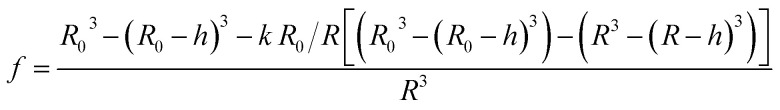
where the expression in the brackets is referring to the difference in lipid fraction of the initial volume and the shrunken vesicle. The prefactor *k* is the fraction of the time dependent ratio *R*_0_/*R*. The results summarized in Fig. 15 illustrate that it is indeed possible to reproduce the second kinetic seen in scattering experiments where we exposed OG containing vesicles to a hyperosmotic gradient with reduced [OG].

## 4. Discussion

### 4.1. Hyper-/hypoosmotic conditions

**Fig. 16:**
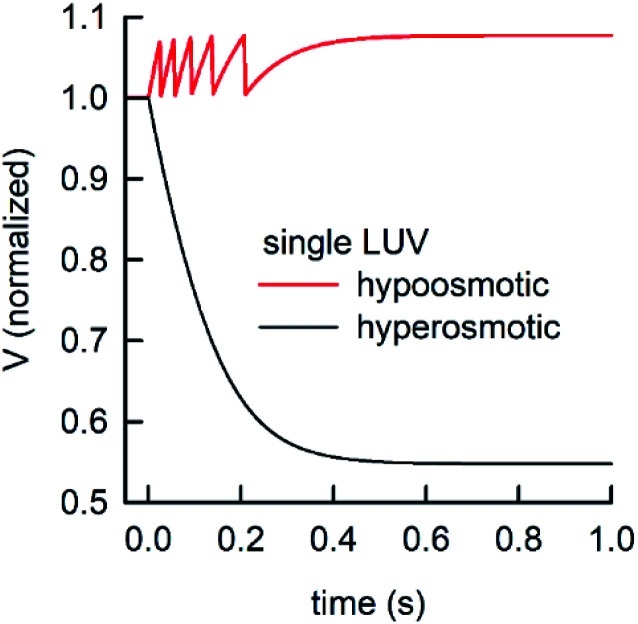
Normalized vesicle volume after subjection to a hyper- or hypoosmotic solution. The red line illustrates that during each swelling–burst cycle the vesicle volume increases until the maximum sustainable strain is reached. Further influx of water leads to rupture of the membrane, an outflux of water and solute, vesicle shrinkage and membrane recovery, which is illustrated by the falling flank of this saw tooth like part of the signal. The swelling–burst cycle repeats until the final plateau is reached beyond the maximum volume change.

LUVs or GUVs are perfect osmosensors. This osmotic sensitivity is lost in the case of very small vesicles obtained by extensive sonication.^79^ Hereby, the osmotic pressure seems not enough to overcome the tension in the highly curved phospholipid bilayer. The lower size limit of such vesicles is envisioned to depend on the phospholipid composition of the bilayer and is approximately 10 nm in radius for synthetic phosphatidylcholines with varying chain lengths.^80^ For larger vesicles hypoosmotic gradients cause vesicle swelling which results in processes strongly determined by vesicle rupture and content efflux. There is no physical basis to fit scattering or self-quenching data at hypoosmotic conditions with equations as described in this paper. Published differences in *P*_f_ between hyper- and hypoosmotic conditions^23^ can solely be explained by the improper measurement conditions and data evaluation procedure. Our experiments with PLE vesicles at room temperature show that the membrane integrity is lost above 3.2% volume increase or 2.1% area expansion. This is in reasonable agreement to an average maximum increase in vesicle volume until the membrane ruptures of about 8%^81^ or 5%^76^ for 1-palmitoyl-2-oleoyl-*sn-glycero*-3-phosphocholine (POPC) and lecithin GUVs, respectively. Fig. 16 illustrates that, in contrast to hyperosmotic conditions where *V*(*t*) decreases with time (black solid line), the vesicle undergoes multiple swelling–burst cycles under hypoosmotic conditions (red solid line). Such swelling–burst cycles could be directly visualized for GUVs,^81^ however at longer time scales due to their larger vesicle volume compared to LUVs.

Clearly, these swelling–burst cycles cannot be directly visualized for LUVs in ensemble measurements. On average, they lead to scattering and self-quenching signals which on the first glance look like meaningful measurements under hyperosmotic conditions, but with inverted sign (Fig. S7†). However, at a second glance it is obvious that these kinetics do not represent the kinetics of water flux through the membrane barrier, but are limited by several unrelated parameters including the exact physical properties of the membrane that determine the exact rupture point after vesicle area expansion and the healing time as well as the buffer exchange between the vesicle interior and exterior during vesicle rupture. However, the fluorophore release assay^82^ performed in Fig. 10, as indication for membrane leakage, can be used to identify the maximum area expansion (2.1% for PLE) of such membranes before rupture. Moreover, this assay is suitable to study the process of membrane healing in more detail in the future. It is important to note that the accuracy of membrane expansion measurements is much higher with the micropipette pressurization method which also allows to verify elastic reversibility.^83^

### 4.2. Influence of the fluorophore concentration in self-quenching experiments on *P*_f_

The pH dependent fluorescence properties of CF, a dye typically used for self-quenching experiments, are used to relate the changes of *F*(*t*) at low [CF] (∼0.5 mM) to proton and weak acid permeabilities.^41,84,85^ Usually, in such experiments with [CF] in the low mM range the self-quenching properties are neglected.^86^ However, our results clearly show that depending on the accompanying volume changes it is necessary to consider them also in pH experiments. Additionally, low [CF] can be used to measure both, volume changes for water flux and pH changes for proton and weak acid permeabilities with one batch of PLs. The accompanying error in *P*_f_ estimation depending on the fluorophore concentration is moderate (maximal 25%). Nevertheless, with eqn (S1) and (S2)† we herein provide a correction term for the fluorophore concentration used.

### 4.3. Vesicle size distribution

*P*_f_ linearly scales with the error in *r*_0_.^18^ It is justifiable to use the average radius of the unimodal size distributions to accurately calculate *P*_f_ values from *I*(*t*) and *F*(*t*) ensemble stopped-flow data. The average radius can either be recalculated from the scattering distribution from DLS measurements or extracted from FCS measurements. The average hydrodynamic radius accessible with FCS, is most closely related to the average radius of the volume distributions of DLS (see Materials and methods section *Vesicle size distribution/average mean diameter* for more details). Average radii from FCS cannot be converted to average diameters of the intensity or volume weighted distributions as the volume distribution is not accessible by FCS but only the mean radius. This average radius of the volume weighted distribution is important for the evaluation of self-quenching data. In contrast, for scattering experiments the average radius of the intensity distribution has to be used. There is no wavelength correction for the different wavelengths used in DLS and scattering experiments necessary, since there is hardly any wavelength dependence as exemplified in Fig. S4.† Obviously, these considerations cannot be directly translated to the problem of polydisperse distributions even though the considerations herein might suggest that an average radius might also be a good approximation for such experimental conditions.

### 4.4. Variability of *P*_f_ in a vesicle population

MP reconstitution does not result in a homogeneous distribution of MPs between all PLs, but the optimization of the reconstitution efficiency leads to a fraction of empty vesicles^2,87^ and a distribution of MPs within the fraction of PLs.^19^ Hence, *I*(*t*) and *F*(*t*), resemble a superposition of different *P*_f_ values from vesicles with varying amounts of artificial channels, channel forming peptides, or MPs and a fraction of bare lipid vesicles. Whereas, the bare lipid vesicle fractions can be described by a single *P*_f_ value, in reality the ensemble of PLs has to be described by a distribution of *P*_f_ values, corresponding to the distribution of MPs in the PLs. To estimate the effect of this distribution on *P*_f_ values we simulated a realistic reconstitution series of AQP1 in PLE, assuming varying average amounts of Poisson distributed AQP1. The *in silic*o experiments clearly show that the error is neglectable as soon as the distribution of the number of channels in the vesicle ensemble is symmetric. In case the distribution is asymmetric as in the case of *N*_mean_ < 5 for a Poisson distribution the error in *P*_f_ estimated from an overall fit to the data compared to the *P*_f_ used for the simulation approaches 100%. Similarly important is a high fraction of protein containing vesicles as compared to empty vesicles. To keep the ‘empty’ liposome fraction below 0.5 and increase *N*_mean_ above 5 it may be necessary to enhance the reconstitution efficiency. Parameters which can be tuned and optimized are the choice of the right detergent, the initial protein to lipid ratio (and concentration), detergent removal (removal speed, temperature) and the buffer composition. The bare lipid vesicle fraction can be determined using FCS to compare the total number of vesicles (vesicles contain labelled lipids, *e.g.* red labelled) with the ones that contain protein (*e.g.* protein is labelled with a green fluorophore).^1,2,19,20^ A similar strategy can be used to accurately quantify protein abundance *N*_mean_.^42^ Thereby the number of PLs containing labeled protein are compared to the number of individual protein oligomer containing micelles after detergent addition.

### 4.5. Residual detergent after protein reconstitution

During reconstitution of membrane proteins into lipid vesicles or LPSs membrane proteins are mixed with detergent and the membrane matrix material. To induce vesicle formation detergent is removed either by dialysis,^88^ addition of BioBeads,^89,90^ dilution^88^ or cyclodextrins.^91^ However, due to variable detergent affinities to BioBeads and proteins and different critical micellar concentrations of detergents, complete removal of used detergents poses a challenge and is hard to detect. We therefore investigated the influence of residual detergent after MP reconstitution on the example of the widely used detergent octyl glucoside (OG). However, we want to emphasize that detergent effects on scattering and self-quenching data in general and on *P*_f_ in particular depend on the experimental conditions. The membrane-water partition coefficient of detergent molecules as well as the flip-flop from one monolayer into the other monolayer strongly vary for different detergents and lipids.^92^ The results show that the presence of OG caused a relative increase in *P*_f_ of less than 60–70% depending on the osmolyte used. Therefore, we suggest performing water flux measurements always in a MP concentration dependent manner to reduce the unknown detergent effects in the preparation. Assuming that (i) detergent removal is similar between multiple samples in one preparation and (ii) it is independent on the protein concentration due to the negligible stoichiometry ratio of protein to detergent molecules, a potential detergent effect incurs in the bilayer background permeability from a fit to a *P*_f_ over *N*_mean_ plot.

Most interestingly, dilution of OG in the measurement cuvette leads to a temperature dependent extraction of OG out of the lipid bilayer into the buffer visible as a second kinetic in the scattering data, which is sensitive to the refractive index of the vesicle defined by the refractive indices and the respective volume fractions of the membrane and the interior. In contrast, application of a hyperosmotic gradient did not influence fluorescence self-quenching experiments as the process of detergent extraction, in turn decreasing the vesicle membrane area does only change the vesicle shape towards a sphere but doesn't affect the volume change which is solely defined by the osmotic gradient. At this stage it remains unclear if the rapid withdrawal of OG from the outer vesicle leaflet creates a mass imbalance between both leaflets resulting in a destabilized inner leaflet forming mixed micellar structures within the inner monolayer or if flip-flop of OG between both monolayers occurs on a similar timescale as the detergent partitioning into the aqueous phase.^93^ Hence, stopped-flow light scattering experiments are perfectly capable of monitoring detergent removal from lipid- or polymer-based vesicles as well as to proof the presence of detergent remaining in vesicular membrane systems.

## 5. Conclusion

For most applications, fluorescence self-quenching and light scattering experiments are equally well capable of delivering accurate *P*_f_ values if performed correctly. However, fluorescence self-quenching experiments are more robust and less prone to artefacts as they solely depend on the vesicle volume as the sole measurement parameter. Therefore, interference from the lipid or polymer membrane, vesicle shape or deformation and extraction of detergent from lipid vesicles can be neglected. The only drawback is that they involve two more steps during sample preparation; (i) addition of the dye during vesicle formation and (ii) removal of free dye before the experiment. Furthermore, the fluorophore used should not interact with any substance in the experiment that lead to a change in fluorescent properties. Light scattering on the other hand has an additional sensitivity towards refractive index changes, which are temperature, wavelength and concentration dependent. This on the first glance advantages property for solute permeability measurements leads to a reduced sensitivity as the refractive index of the particle is dominated by the refractive index of the membrane matrix and not the interior, which significantly reduces its amplitude compared to pure volume changes.

To ensure accurate *P*_f_ estimations it is necessary to calculate the average radius of the relevant vesicle size distribution, ensure a high reconstitution efficiency of MPs into PLs, use the models described herein (either analytical solution or approximation based on an exponential fit), use hyperosmotic measurement conditions, and avoid residual detergent in vesicles. In light scattering experiments lower excitation wavelengths and osmolytes exhibiting a large refractive index compared to the buffer solution maximize the relative signal amplitude. Yet, care has to be taken to ensure the applicability of the RGD relation, which was the case for all *in silico* and *in vitro* experiments performed throughout this study. For further estimations of *p*_f,_ it is inevitable to correlate the stopped-flow data with accurate channel counting. The accuracy of *p*_f_ estimations increases significantly using reconstitution series with varying amounts of channels in the PLs and using a global fitting routine were the *P*_f_ of the bare lipid vesicles fraction is fixed. A linear dependence of *P*_f_ on the average number of channels per vesicle will help to dispel any doubts on the significance of *p*_f_ values which can be directly calculated from the slope of a linear regression to the data points.

## Author contributions

A. H. conceived the project. J. W., A. S., T. B and A. E. performed stopped-flow and DLS experiments. T. B. performed FCS experiments. J. W. and A. S. performed simulations. J. W., C. H., A. S., T. B, A. E. and A. H. analyzed *in vitro* and/or *in silico* data. J. W. and C. S. expressed, purified and reconstituted AQP1. J. W. and A. H. wrote the manuscript. All authors commented on the manuscript.

## Conflicts of interest

There are no conflicts to declare.

## Supplementary Material

NA-004-D1NA00577D-s001
