# Supplementary material for: Scattering versus fluorescence self-quenching: more than a question of faith for the quantification of water flux in large unilamellar vesicles?
Source: Nanoscale Adv. 2021 Oct 18;4(1):58–76. doi: 10.1039/d1na00577d (PMC8691418; doi:10.1039/d1na00577d)
Supplement: NA-004-D1NA00577D-s001 [file NA-004-D1NA00577D-s001.pdf]

Electronic Supplementary Material (ESI) for Nanoscale Advances.
This journal is © The Royal Society of Chemistry 2021

Supplementary Information

Scattering versus Fluorescence Self-Quenching: More than a Question of Faith for the Quantification of Water Flux in Large Unilamellar Vesicles?

Johann Wachlmayr, Christof Hannesschlaeger, Armin Speletz, Thomas Barta, Anna Eckerstorfer, Christine Siligan and Andreas Horner[#]

From the Institute of Biophysics, Johannes Kepler University Linz, Gruberstr. 40, 4020 Linz, Austria; [#]Correspondence should be sent to andreas.horner@jku.at

Fitting routine	P_f (µm/s)	Error (%)
Exp 1st order	6.543	9.05
Exp 2nd order	7.816	30.27
Ana 1st order	6.445	7.42
Ana 2nd order	6.445	7.42

Table S1. P_f values and errors. *The simulated scattering curve, shown in **Figure 4** was fitted with a mono exponential (Exp) and with the analytical solution (Ana), each one using a Taylor approximation for the I(t)-V(t)-relationship until 1st and 2nd order, respectively.*

A

WL (nm)	P_f (µm/s)	Error (%)
346	6.336	5.59
446	6.408	6.80
546	6.445	7.42
646	6.531	8.85
846	6.606	10.09

B

	Simulation		Measurement	
WL (nm)	P_f (µm/s)	Error (%)	P_f (µm/s)	Error (%)
313	8.85	0.98	7.39	4.64
405	8.85	0.95	7.30	3.28
546	8.94	0.00	7.06	0.00

Table S2. Excitation wavelength influences I(t). *(A) P_f values and errors of fitted simulations of scattered light intensity traces (see **Figure 5A**) upon exposure to a hyperosmotic gradient for different wavelengths WL of the illuminating monochromatic light. (B) P_f values and errors of fitted simulated and experimental scattering data (see **Figure 5A**) for different wavelength. The simulation conditions for buffer osmolarities and vesicle size distribution have been adapted to the experimental conditions (192 mOsm NaCl, 10 mOsm MOPS, 148 mOsm sucrose, $R_{vesicle}$ = 55.75 nm).*

d_{mem} (nm)	P_f (µm/s)	Error (%)
1	5.993	0.11
2	6.275	4.58
4	6.445	7.42
8	6.861	14.34
15	7.788	29.80
30	10.980	83.01

Table S3. Effect of membrane thickness on P_f. *P_f values and errors of the simulated scattering curves with varying membrane thickness, shown in **Figure 7**.*

		P_f				
	T (°C)	0.00 % OG	0.01 % OG	0.03 % OG	0.1 % OG	0.3 % OG
Scattering	4	6.72	6.52	7.02	7.30	6.98
	14	13.14	13.60	14.49	14.45	11.21
	24	26.90	30.01	33.16	34.90	24.77
	34	56.22	63.93	74.33	34.68	24.66
self-quenching	4	6.08	6.16	6.36	7.03	6.14
	14	14.71	14.93	15.42	16.89	13.87
	24	31.11	31.60	37.67	36.88	30.18
	34	65.70	64.37	65.46	36.19	29.13

Table S4. *P_f values for scattering and self-quenching traces at different detergent concentrations and temperatures after subjection to a hyperosmotic solution. The curves of **Figure S13** have been fitted with an exponential with 2 free components, except for self-quenching mode and the sample without detergent, which show no second component.*

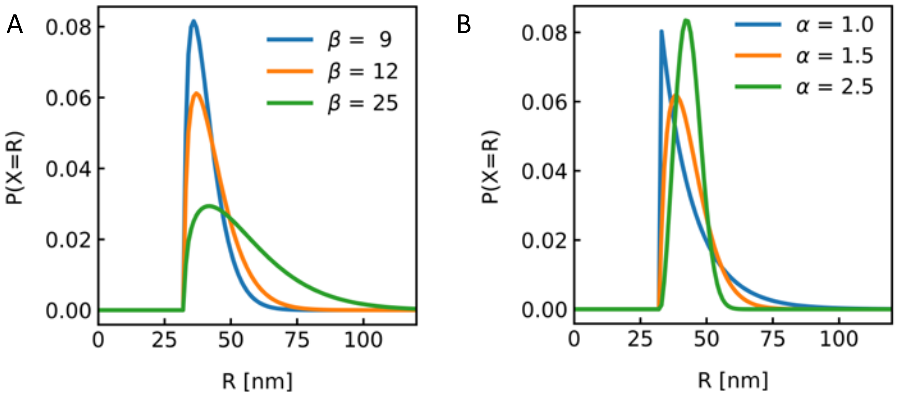

Figure S1. Probability density function of the Weibull distribution*; The Weibull parameters used for simulations are except explicitly mentioned: α = 1.35, β = 12.1, γ = 32.6. (A) Variation of β. (B) Variation of α.*

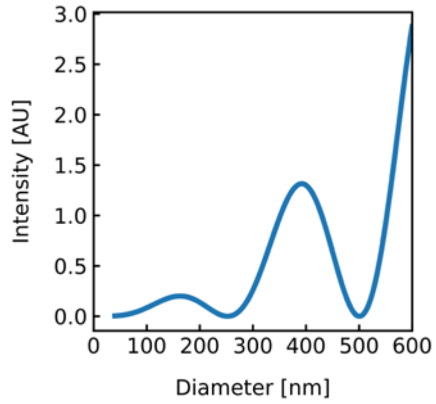

Figure S2. Scattering intensity at a detection angle of 165° over vesicle size. *The RGD theory reveals 'blind spots', where vesicles with 250 nm and 500 nm cannot be detected.*

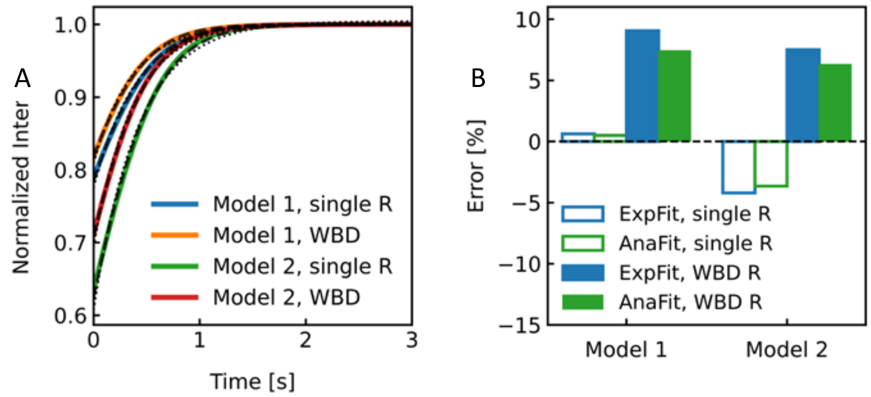

Figure S3. Effect of vesicle form parameter $P(\theta)$ on I(t). *(A) The Homogeneous Sphere Model (Model 1) with single radius (blue) and Weibull distributed (WBD) vesicle sizes (orange) are compared to the Hollow Sphere Model (Model 2 with single radius (green) and WBD vesicle sizes (red)). Dashed and dotted black lines represent the analytical and exponential fits, respectively. (B) Errors in P_f of the 2 models simulated with single radius (empty bars) and Weibull distributed radii (filled bars). P_f values are obtained from fits with a mono exponential function (blue) and the analytical solution (green).*

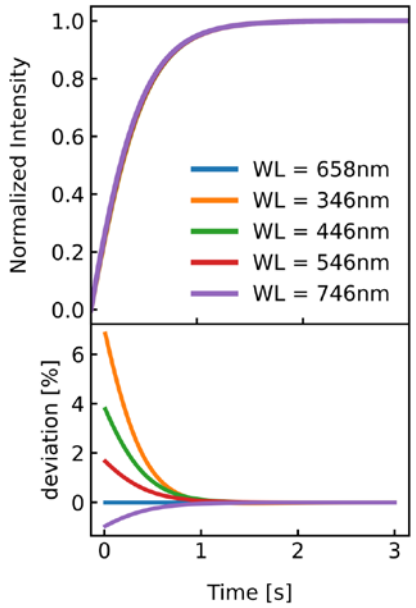

WL (nm)	Pf (µm/s)	Error (%)
658	6.485	0.00
346	6.385	1.55
446	6.409	1.19
546	6.446	0.61
746	6.515	0.45

Figure S4. Scattering traces of hyperosmotic shrinkage simulations. *P_f values and errors for different excitation wavelengths. The deviation of the different curves to the simulation at 658 nm wavelength of the incident light is depicted below. As the differences in P_f values and the deviation of the curves are small, the error of measuring the size of the vesicles by DLS at 658 nm and performing stopped flow experiments at different wavelengths can be neglected.*

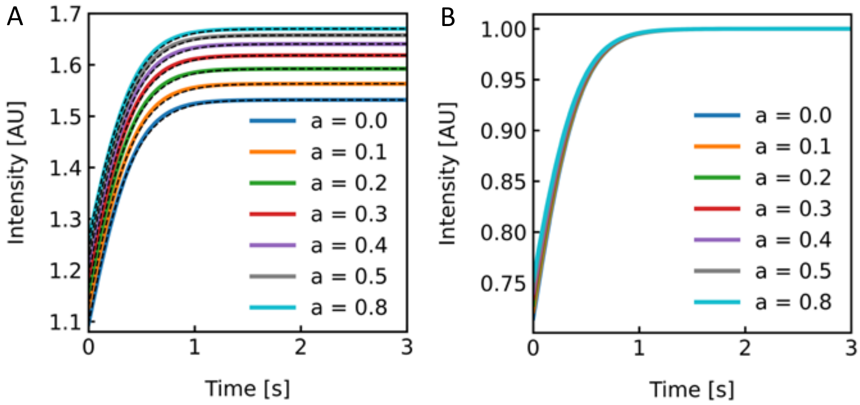

Figure S5. Simulated scattering signal for hyperosmotic shrinkage curves of vesicles with increasing ellipticity. *Vesicles with length $l_a = R_0 + aR_0$ of the vesicle long axis and length $l_b = R_0 - aR_0$ of the vesicles short axis have been simulated. A value of a = 0.0 (blue curve) therefore corresponds to a spherical vesicle, and a = 0.8 (cyan) represents the vesicle with the highest ellipticity (ratio of l_a to l_b). The scattering traces are plotted in absolute intensities (in arbitrary units) (A) and normalized (B). The error in P_f when fitted with the analytical solution was < 1.5% and can thus be neglected.*

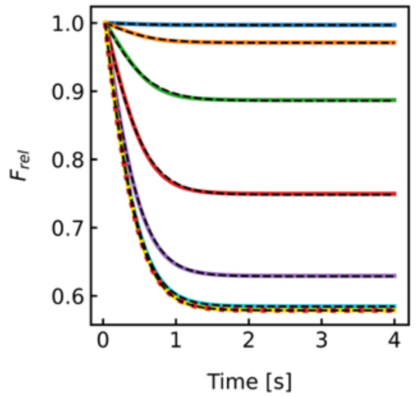

Figure S6. Relative fluorescence of a single fluorophore F_{rel} over time. *The time dependent volume change and the corresponding change in fluorescence signal were simulated according to* ***Eqn. 4*** *and* ***Eqn. 22****, respectively. A fixed radius of 50 nm was used for simulation, all other settings were chosen as described in Figure 1. The resulting curves were fitted with* ***Eqn. 11*** *and the error in P_f is plotted in the inset of* ***Figure 8B****. The relative fluorescence change is shown for different initial fluorophore concentrations corresponding to 0.01 mM (blue curve), 0.1 mM (orange), 0.5 mM (green), 2 mM (red), 10 mM (purple) and 100 mM (cyan). The corresponding corrected curves, according to* ***Eqn. 24****, and the relative volume change are represented by the solid yellow and dotted red line, respectively. All curves have been fitted to the analytical solution (****Eqn. 5*** *and* ***Eqn. 18****).*

The nonlinear dependence of *F(t)* on *V(t)* for low dye concentration can either be corrected by

$$V(t)_{corr} = \frac{F_{rel}(t) K_s V_0 c_{f,0}}{1 + K_s \cdot c_{f,0} - F_{rel}(t)} \qquad \text{S1}$$

where $V(t)_{corr}$ is the corrected volume of the vesicle at time t, or by correcting P_f with the result of the fit (black dashed line) of the error plot (cyan curve in the inset of ***Figure 8B).***

$$P_{f,corr} = \frac{P_{f,fit}}{1 + (A + Be^{-k_1 c_f} + Ce^{-k_2 c_f})} \qquad \text{S2}$$

where $P_{f,corr}$ is the corrected water permeability coefficient. The fitting parameter $A = -1.03$, $B = 7.48$, $C = 15.3$, $k_1 = 57.47$ M^{-1} and $k_2 = 529.56$ M^{-1}. c_f is the initial fluorophore concentration.

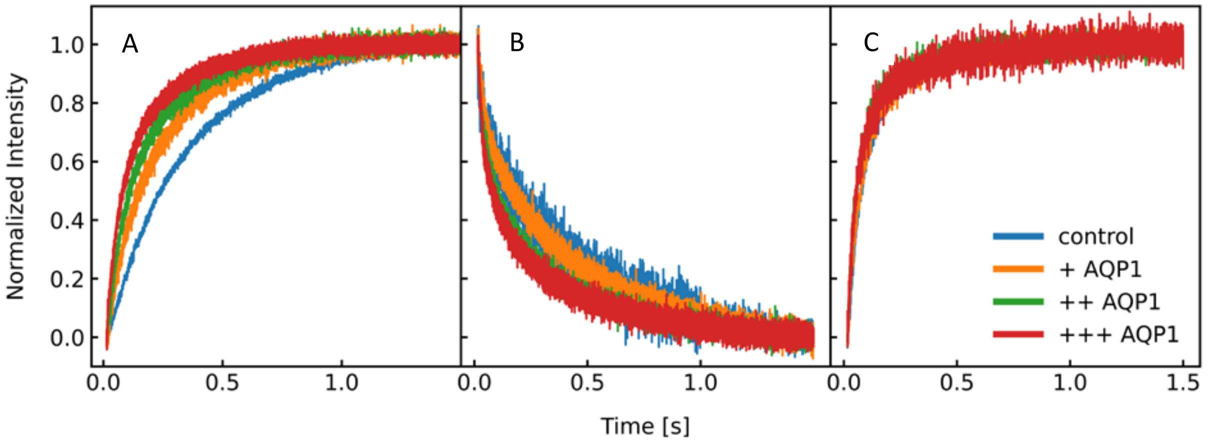

Figure S7. Exemplary scattering and self-quenching curves after subjection to a hyper- and hypoosmotic solution, respectively. *PLE liposomes (blue) and proteoliposomes with increasing amount of incorporated AQP1 (from orange to red) were subjected to an osmotic gradient. Hyperosmotic measurements in scattering mode (A) are compared to hypoosmotic scattering curves (B) and hypoosmotic self-quenching curves (C). The curves have not been fitted, as the incorporated carboxyfluoresceine makes protein counting impossible, which is necessary for the determination of the single channel permeability. In case of hypoosmotic traces, there is no evidence to apply the exponential nor the analytical fit to extract* P_f.

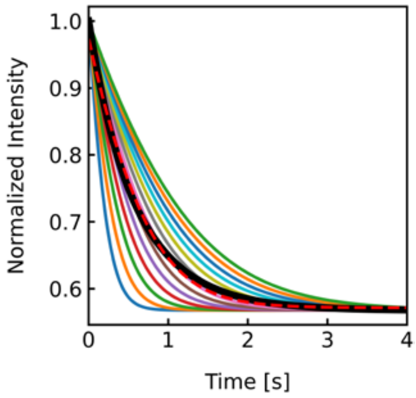

Figure S8. Effect of vesicle size distribution on P_f. *Family of self-quenching curves and their average (black) after exposure to a 150 mosm sucrose gradient. The initial radii of the vesicles were simulated in 10 nm steps ranging from 30 nm (blue) to 150 nm (green). In this case each radius has a similar occurrence in the population similar to* ***Figure 10A*** *in the main text for scattering data. The error in* P_f *between simulation and analytical fit was ~ 4 %.*

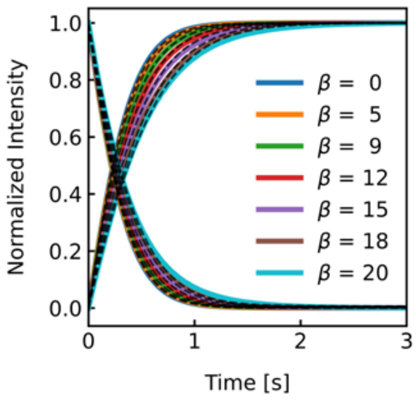

		scattering		self-quenching	
β	R_{INT} (nm)	P_f (µm/s)	Error (%)	P_f (µm/s)	Error (%)
0	43.66	6.23	3.90	6.54	8.98
5	45.28	6.11	1.84	6.49	8.14
9	50.38	6.24	4.02	6.49	8.15
12	56.48	6.38	6.26	6.51	8.47
15	63.67	6.50	8.37	6.53	8.9
18	71.87	6.61	10.18	6.56	9.34

20	77.64	6.67	11.15	6.57	9.51

Figure S9. Variation of β; *Simulation of scattered light intensity traces of Weibull distributed vesicles with varying scale parameter β after exposure to a hyperosmotic gradient. P_f values and errors obtained from fitting the analytical solution to the curves are tabulated. Figure 10B is normalized between 0 and 1 to visualize the different kinetics.*

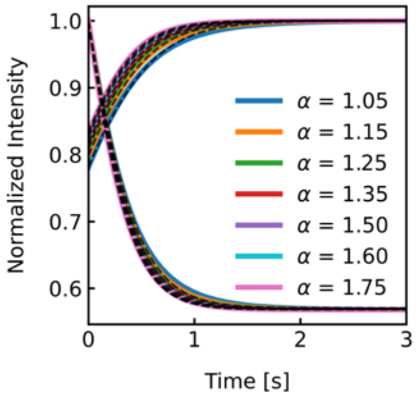

		scattering		self-quenching	
α	R_{NT} (nm)	P_f (µm/s)	Error (%)	P_f (µm/s)	Error (%)
1.05	76.82	7.04	17.28	6.60	10.00
1.15	66.54	6.70	11.69	6.58	9.62
1.25	60.38	6.50	8.35	6.53	8.86
1.35	56.48	6.38	6.26	6.51	8.47
1.50	52.88	6.27	4.50	6.49	8.21
1.60	51.37	6.24	3.94	6.49	8.13
1.75	49.87	6.21	3.57	6.50	8.26

Figure S10. Variation of α; *(A) Simulation of scattered light intensity traces of Weibull distributed vesicles with varying shape parameter α after exposure to a hyperosmotic gradient. While the scale parameter β was kept constant, the location parameter γ was adjusted to achieve an expectation value for the number- weighted radius of 43.7 nm. The conversion of number to intensity distribution was done according to **Eqn. S3** and the resulting distribution was fitted with a Weibull distribution function (**Eqn. 8**) to obtain the intensity- weighted radii R_{INT}. P_f values and errors obtained from fitting the analytical solution to the curves are tabulated.*

$$N_i^I = \frac{N_i^N {r_i}^6}{\sum_i N_i^N {r_i}^6} \tag{S3}$$

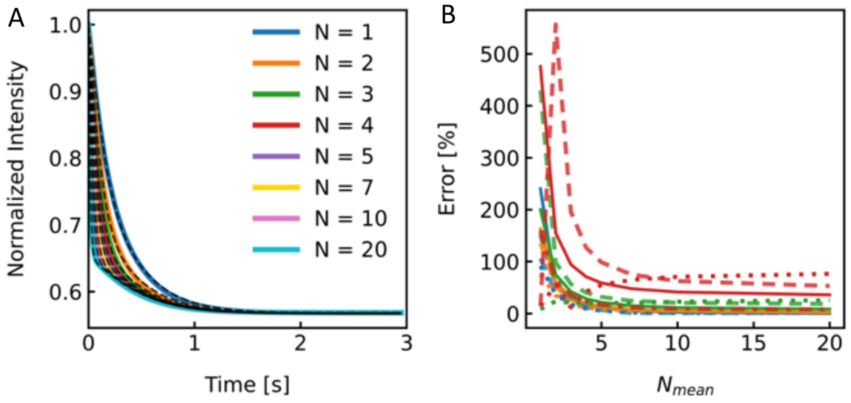

Figure S11. Effect of MP distribution on P_f. *(A) Normalized self-quenching plot of osmotic vesicle shrinkage for PLs of different N_{mean}. The fraction of vesicles which do not contain protein $x_l = 0.2$. (B) Error in P_f depending on N_{mean} for $x_l = 0.0$ (blue), $x_l = 0.2$ (orange), $x_l = 0.5$ (green) and $x_l = 0.8$ (red). Dotted, dashed, dashdotted and solid lines represent the error for a two-component exponential fit with free components, a two-component exponential fit, where one component is fixed to the rate constant of liposomes containing no protein, an analytical fit and a global analytical fit, respectively.*

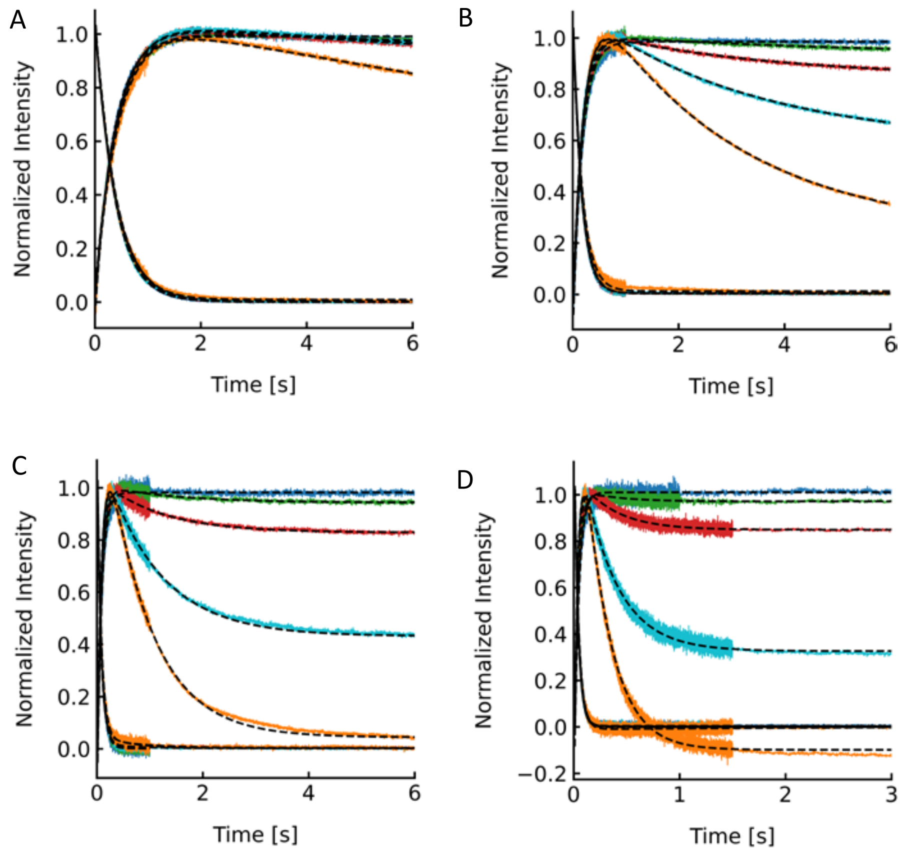

Figure S12. Exemplary averaged scattering and self-quenching traces at different detergent concentrations and temperatures*.* *Samples are subjected to a hyperosmotic solution at 4°C (A), 14°C (B), 24°C (C) and 34°C (D). For each sample, 10 mg PLE was used with 1 ml of buffer containing 10 mM CF, 100 mM NaCl and 10 mM MOPS at pH 7.4 and the OG concentration was 0% (blue curve), 0.01% (green), 0.03% (red), 0.1% (cyan) and 0.3% (orange), respectively.*

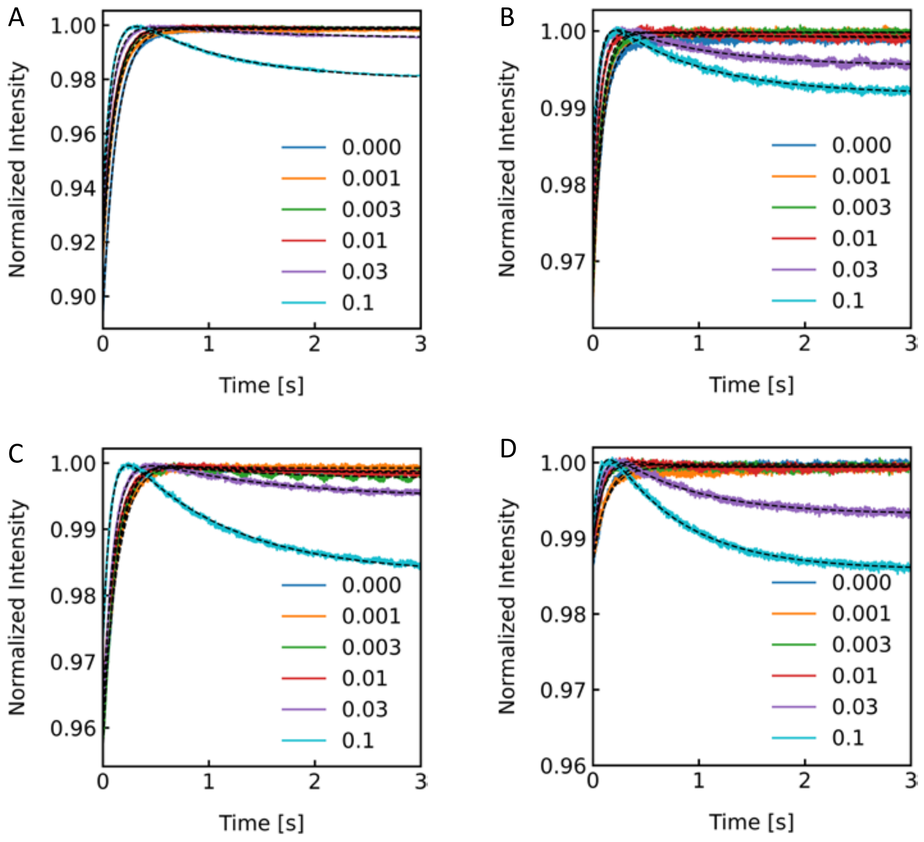

Figure S13. Scattering traces for different detergent concentrations and osmolytes. *PLE liposomes containing 0% (blue curve), 0.001% (orange), 0.003% (green), 0.01% (red), 0.03% (purple) and 0.1% OG (cyan) were exposed to a hyperosmotic gradient (100 mM) of sucrose (A), glucose (B), sodium chloride (C) and urea (D) at a temperature of 20°C. The buffer conditions inside the vesicles were 10 mM NaCl, 1 mM MOPS and 300 mM of the corresponding osmolyte and outside the vesicles, the initial buffer concentrations were 10 mM NaCl, 1 mM MOPS and 400 mM osmolyte.*

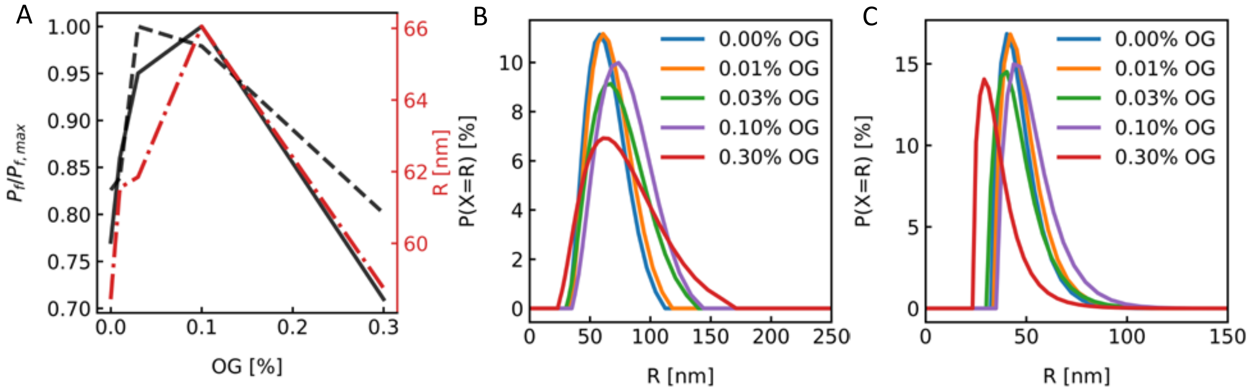

Figure S14. Dependency of permeability coefficient and vesicle size on the amount of residual detergent. *(A) The radius of the vesicles (red, dash-dotted curve) and the normalized permeability coefficients measured in scattering (black solid curve) and self-quenching mode (black dashed curve) are plotted against the OG concentration. (B) Intensity distribution for the vesicles with different amount of OG. (C) Intensity distribution for the vesicles with different amount of OG.*
